# Supplementary material for: Assessment of infant outgrowth of cow’s milk allergy in relation to the faecal microbiome and metaproteome
Source: Sci Rep. 2023 Jul 25;13:12029. doi: 10.1038/s41598-023-39260-w (PMC10368738; doi:10.1038/s41598-023-39260-w)
Supplement: Supplementary file 1 — Supplementary Information 1. [file 41598_2023_39260_MOESM1_ESM.pdf]

Supplementary results for:

Assessment of infant outgrowth of cow's milk allergy in relation to the faecal microbiome and metaproteome

Diana M. Hendrickx[1,*], Ran An[1,+], Sjef Boeren[2], Sumanth K Mutte[2], PRESTO study team[#], Jolanda M Lambert[3], and Clara Belzer[1]

[1]Laboratory of Microbiology, Wageningen University, Wageningen, The Netherlands
[2]Laboratory of Biochemistry, Wageningen University, Wageningen, The Netherlands
[3]Danone Nutricia Research, Utrecht, The Netherlands
* diana.hendrickx@wur.nl; clara.belzer@wur.nl
[+] current address: Department of Food Science and Technology, School of Agriculture and Biology, Shanghai Jiao Tong University, Shanghai 200240, China.
A list of authors and their affiliations appears at the end of the paper.

Table S1. Clinical characteristics. Numeric variables are presented as mean ± standard deviation; categorical variable are presented as number (%). SCORAD: severity of atopic dermatitis. AAF-syn: amino acid-based formula + synbiotics; AAF: standard amino acid-based formula. Vomiting, spitting, stool frequency, stool consistency, stool colour, gas/wind: parent reported outcomes - mean score of 3 reported values. Vomiting: 0 = none; 1 = 1-2 times/day; 2 = 3-4 times/day. Spitting: 0 = none; 1 = spitting up after some feeds; 2 = spitting up after all feeds. Stool frequency: 0 = none; 1 = few; 2 = several; 3 = a lot. Stool consistency: 0 = Severe diarrhoea; 1 = Diarrhoea; 2 = Normal; 3 = Constipation. Stool colour: 0 = green; 1 = yellow; 2 = brown; 3 = Dark brown / Blackish. Gas / wind: 0 = none; 1 = mild; 2 = moderate; 3 = severe.

**characteristic**	**outgrowth of CMA (n=24)**	**no outgrowth of CMA (n=15)**	**total (n=39)**
sex: female	8 (33%)	3 (20%)	11 (28%)
male	16 (67%)	12 (80%)	28 (72%)
age: baseline	8.56 ± 3.04	9.68 ± 2.63	9.00 ± 2.90
6 months	14.62 ± 3.02	15.59 ± 2.54	14.99 ± 2.85
12 months	20.84 ± 3.05	21.88 ± 3.01	21.24 ± 3.03
race: Causasian/white	5 (21%)	4 (27%)	9 (23%)
Asian	18 (75%)	10 (67%)	28 (72%)
Combination of the above/other	1 (4%)	1 (7%)	2 (5%)
mode of delivery:			
vaginal	6 (25%)	7 (47%)	13 (33%)
ceasarian	18 (75%)	8 (53%)	26 (67%)
allergy mother: yes	9 (38%)	10 (67%)	19 (49%)
no	15 (63%)	5 (33%)	20 (51%)
allergy father: yes	6 (25%)	9 (60%)	15 (38%)
no	18 (75%)	6 (40%)	24 (62%)
sibling: yes	18 (75%)	10 (67%)	28 (72%)
no	6 (25%)	5 (33%)	11 (28%)
SCORAD: baseline	8.98 ± 14.41	16.27 ± 13.24	11.78 ± 14.25
6 months	5.46 ± 8.32	8.13 ± 9.67	6.49 ± 8.84
12 months	6.77 ± 8.25	10.37 ± 8.77	8.15 ± 8.52
egg allergy: yes	9 (38%)	5 (33%)	14 (36%)
no	15 (63%)	10 (67%)	25 (64%)
other food allergy than CMA and egg allergy:			
yes	3 (13%)	6 (40%)	9 (23%)
no	21 (88%)	9 (60%)	30 (77%)
treatment: AAF-syn	14 (58%)	9 (60%)	23 (59%)
AAF	10 (42%)	6 (40%)	16 (41%)
vomiting:			
6 months	0.13 ± 0.29	0.16 ± 0.52	0.14 ± 0.39
12 months	0.04 ± 0.20	0.20 ± 0.43	0.10 ± 0.32
spitting:			
6 months	0.13 ± 0.27	0.11 ± 0.43	0.12 ± 0.34
12 months	0.04 ± 0.15	0.00 ± 0.00	0.03 ± 0.12
stool frequency:			
6 months	1.03 ± 0.24	1.02 ± 0.96	1.03 ± 0.35
12 months	1.11 ± 0.60	0.48 ± 0.17	1.05 ± 0.49
stool consistency:			
6 months	1.60 ± 0.61	1.89 ± 0.84*	1.69 ± 0.70*
12 months	1.92 ± 0.62*	1.97 ± 0.77*	1.94 ± 0.67**
stool colour:			
6 months	1.44 ± 0.61	1.06 ± 0.96*	1.32 ± 0.92*
12 months	1.30 ± 0.80*	1.89 ± 0.78*	1.52 ± 0.83**
gas / wind:			
6 months	0.79 ± 0.61	0.56 ± 0.63	0.70 ± 0.73
12 months	0.86 ± 1.61	0.56 ± 0.63	0.74 ± 0.62
number of antibiotics until visit:			
6 months	0.63 ± 2.61	0.53 ± 1.41	0.59 ± 1.21
12 months	1.25 ± 3.61	2.40 ± 2.64	1.69 ± 2.12
number of infections until visit:			
6 months	1.25 ± 4.61	1.67 ± 1.45	1.41 ± 1.58
12 months	1.96 ± 5.61	3.00 ± 2.30	2.36 ± 2.31

*: 3 missings; **: 6 missings

Table S1. Clinical characteristics (continued)

characteristic	outgrowth of CMA (n=24)	no outgrowth of CMA (n=15)	total (n=39)
study centre:			
Southampton General Hospital (UK)	1 (4%)	0 (0%)	1 (3%)
Charité Hospital Berlin (Germany)	1 (4%)	1 (7%)	2 (5%)
St.-Marien Hospital (Germany)	3 (13%)	1 (7%)	4 (10%)
Ruhr-Universität Bochum im St. Josef-Hospital (Germany)	1 (4%)	0 (0%)	1 (3%)
KK Women's & Children's Hospital (Singapore)	2 (8%)	5 (33%)	7 (18%)
King Chulalongkorn Memorial Hospital (Thailand)	6 (25%)	4 (27%)	10 (26%)
Ramathibodi Hospital (Thailand)	5 (21%)	2 (13%)	7 (18%)
Prince of Songkla Hospital (Thailand)	4 (17%)	1 (7%)	5 (13%)
Texas Children's Hospital (USA)	1 (4%)	1 (7%)	2 (5%)
CM-specific IgE (kU/L)			
baseline	1.70 ± 2.80	23.28 ± 50.99	10.00 ± 32.80
12 months	1.11 ± 1.74	16.88 ± 20.91	7.18 ± 14.94
total IgE (kU/L)			
baseline	144.64 ± 221.27	564.78 ± 831.55	306.23 ± 572.07
12 months	190.87 ± 199.50	562.64 ± 536.20	333.86 ± 404.46

Table S2. List of proteomes used for the proteomics database construction. The last two columns show the SILVA 138 genus and the subtotal of the relative abundances at genus level. For the taxa indicated in red, only the identified proteins (instead of the complete proteomes) were included.

See Supplementary_Table_S2.xlsx

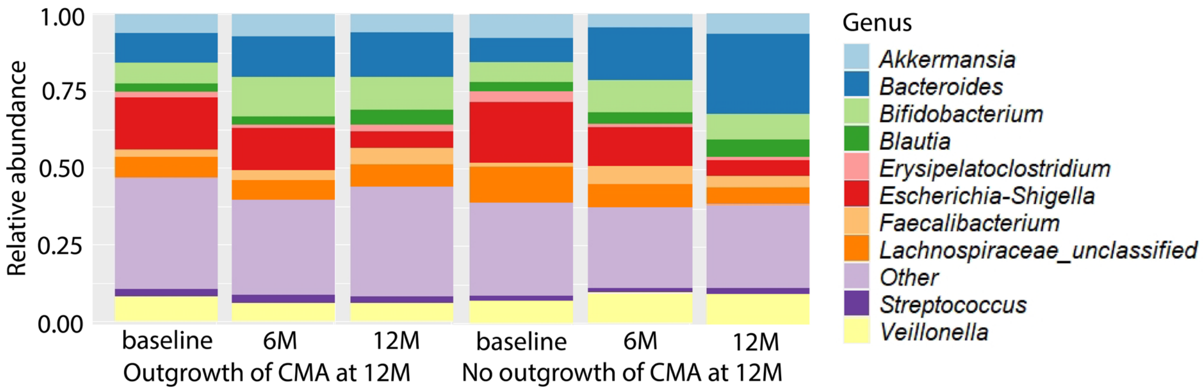

Figure S1. 16S rRNA gene-based taxonomic profiles at the genus level at each visit for the group that outgrew their CMA at visit 12 months (12M) and the group that did not.

Table S3. Clinical factors associated with outgrowth of cow's milk allergy as determined by a two-sided Mann-Whitney U-test for numeric variables and a Fisher's exact test for binary variables. Numeric variables are presented as mean ± standard deviation; categorical variable are presented as number (%). *: 3 missings

**variable**	**outgrowth of CMA (n=24)**	**no outgrowth of CMA (n=15)**	**p-value**
age at baseline	8.56 ± 3.04	9.68 ± 2.63	0.254
allergy of at least one of the parents	yes: 13 (54%)<br>no: 11 (46%)	yes: 13 (87%)<br>no: 2 (13%)	**0.045**
mode of delivery	vaginal: 6 (25%)<br>ceasarian: 18 (75%)	vaginal: 7 (47%)<br>ceasarian: 8 (53%)	0.185
egg allergy	yes: 9 (38%)<br>no: 15 (63%)	yes: 5 (33%)<br>no: 10 (67%)	1.000
gaswind 6 months	0.79 ± 0.61	0.56 ± 0.63	0.504
gaswind 12 months	0.86 ± 1.61	0.56 ± 0.63	0.103
number of antibiotics until visit 6 months	0.63 ± 2.61	0.53 ± 1.41	0.389
number of antibiotics until visit12 months	1.25 ± 3.61	2.40 ± 2.64	0.212
number of infections until visit 6 months	1.25 ± 4.61	1.67 ± 1.45	0.176
number of infections until visit 12 months	1.96 ± 5.61	3.00 ± 2.30	0.096
other food allergy	yes: 3 (13%)<br>no: 21 (88%)	yes: 6 (40%)<br>no: 9 (60%)	0.063
SCORAD 0 months	8.98 ± 14.41	16.27 ± 13.24	**0.036**
SCORAD 6 months	5.46 ± 8.32	8.13 ± 9.67	0.338
SCORAD 12 months	6.77 ± 8.25	10.37 ± 8.77	0.218
sex	female: 8 (33%)<br>male: 16 (67%)	female: 3 (20%)<br>male: 12 (80%)	0.477
sibling	yes: 18 (75%)<br>no: 6 (25%)	yes: 10 (67%)<br>no: 5 (33%)	0.718
spitting 6 months	0.13 ± 0.27	0.11 ± 0.43	0.441
spitting 12 months	0.04 ± 0.15	0.00 ± 0.00	0.274
stool colour 6 months	1.44 ± 0.61	1.06 ± 0.96*	0.096
stool colour 12 months	1.30 ± 0.80*	1.89 ± 0.78*	0.144
stool consistency 6 months	1.60 ± 0.61	1.89 ± 0.84*	0.171
stool consistency 12 months	1.92 ± 0.62*	1.97 ± 0.77*	0.825
stool frequency 6 months	1.03 ± 0.24	1.02 ± 0.96	0.130
stool frequency 12 months	1.11 ± 0.60	0.48 ± 0.17	0.636
treatment (synbiotics vs non-synbiotics)	AAF-syn: 14 (58%)<br>AAF: 10 (42%)	AAF-syn: 9 (60%)<br>AAF: 6 (40%)	1.000
vomiting 6 months	0.13 ± 0.29	0.16 ± 0.52	0.854
vomiting 12 months	0.04 ± 0.20	0.20 ± 0.43	0.125
study centre (western vs non-western country)	western: 7 (29%)<br>non-western: 17 (71%)	western: 3 (20%)<br>non-western: 12 (80%)	0.711

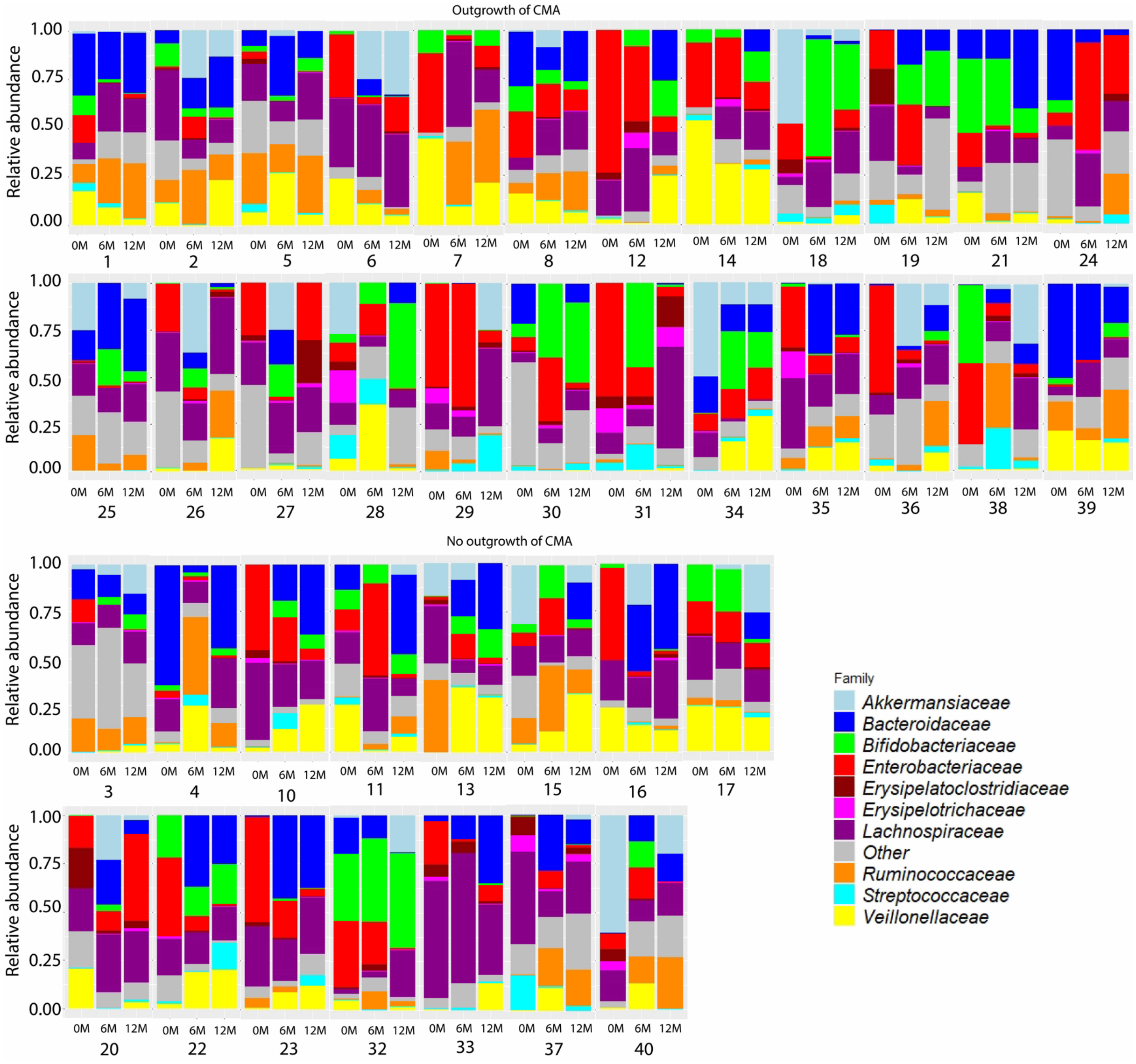

Figure S2. Microbiota composition profiles for each visit (0 months, 6 months, 12 months) for each infant. Taxonomy at family level based on 16S rRNA gene sequencing data.

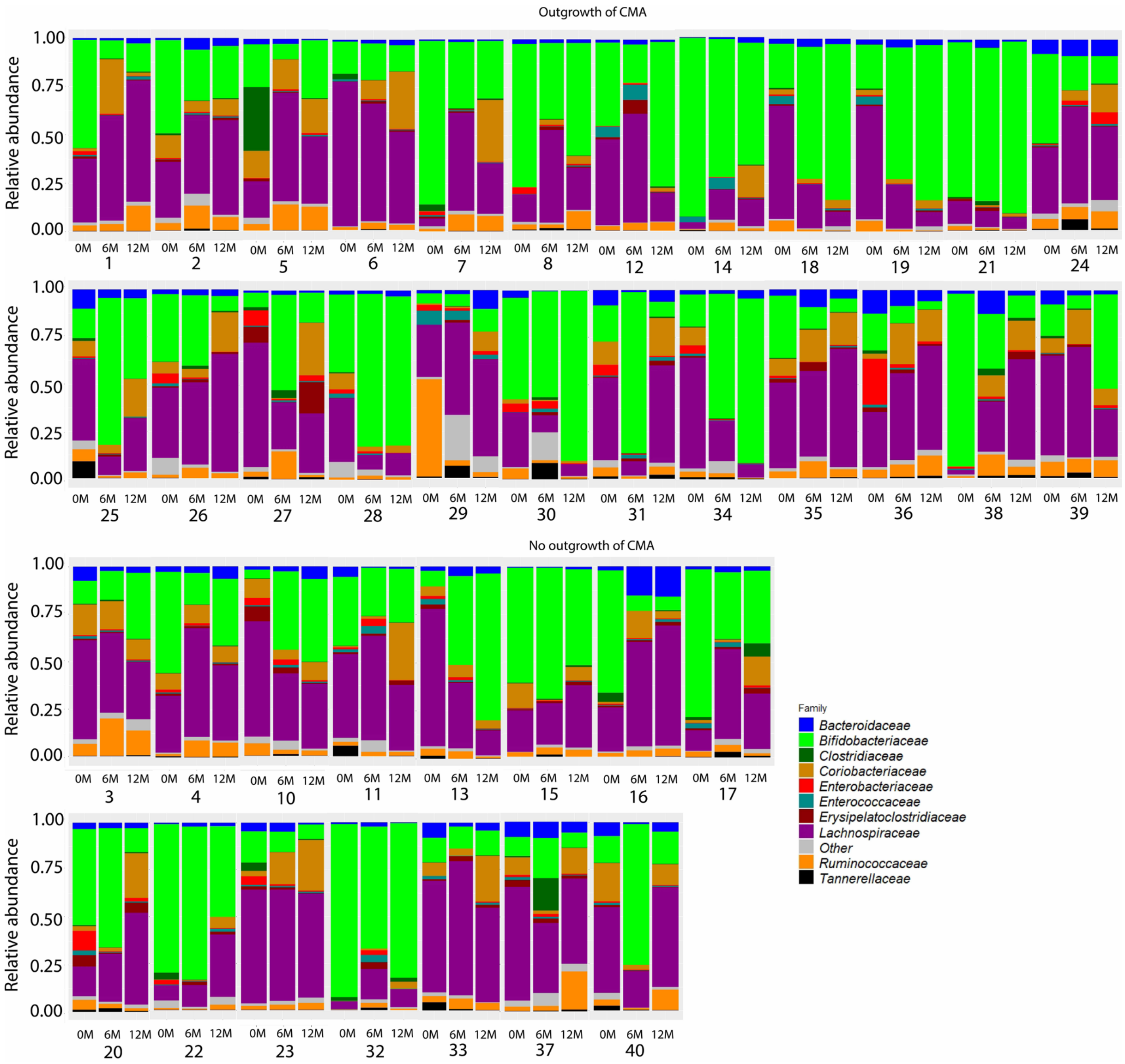

Figure S3. Microbiota composition profiles for each visit (0 months, 6 months, 12 months) for each infant. Taxonomy at family level based on metaproteomics data.

Table S4. Akaike Information Criterion (AIC) values for Linear Mixed Models (LMM) fitted to 16S rRNA gene based relative abundance of core families. Model A: model without age as fixed effect, model B: model including age as fixed effect. For each core family, the best model (lowest AIC) is indicated in bold.

	AIC – model A	AIC – model B
Bifidobacteriaceae	**575.2536**	579.3477
Bacteroidaceae	**548.7505**	549.9798
Lachnospiraceae	**373.4082**	378.1709
Ruminococcaceae	**512.6130**	516.0835
Coriobacteriaceae	**588.2699**	592.4549
Veillonellaceae	**535.6380**	539.9659
Enterobacteriaceae	**456.1456**	457.4884

Table S5. Median 16S rRNA gene based relative abundance and interquartile range (IQR) of core families in faeces samples at baseline and follow-up visits (6 months, 12 months) in infants that outgrew their CMA after 12 months (12M) and children that did not. adj. p-value: p-value determined by Linear Mixed Model (LMM) analysis with Benjamini-Hochberg correction for multiple testing. Core taxa were defined as taxa that have a relative abundance higher than 1% in at least 50% of the 16S rRNA gene or metaproteomics samples. A: model without age as fixed effect, B: model including age as fixed effect.

	**Baseline visit**		
	**Outgrowth of CMA**	**No outgrowth of CMA**	**adj. p-value**
Bifidobacteriaceae	0.025 (0.001-0.079)	0.004 (0.001-0.076)	A: 0.9594; B: 0.9026
Bacteroidaceae	0.004 (0.000-0.166)	0.005 (0.000-0.081)	A: 0.9594; B: 0.9026
Lachnospiraceae	**0.116 (0.058-0.198)**	**0.212 (0.165-0.306)**	**A: 0.0280; B: 0.0457**
Ruminococcaceae	0.004 (0.000-0.070)	0.004 (0.000-0.044)	A: 0.9704; B: 0.9026
Coriobacteriaceae	0.00003 (0.00000-0.00406)	0.00000 (0.00000-0.00022)	A: 0.9594; B: 0.9026
Veillonellaceae	0.023 (0.002-0.154)	0.026 (0.006-0.128)	A: 0.9704; B: 0.9026
Enterobacteriaceae	0.218 (0.084-0.350)	0.165 (0.074-0.373)	A: 0.9594; B: 0.9026
	**Visit 6 months**		
	**Outgrowth of CMA**	**No outgrowth of CMA**	**adj. p-value**
Bifidobacteriaceae	0.059 (0.011-0.195)	0.087 (0.018-0.143)	A: 0.7222; B: 0.6813
Bacteroidaceae	0.084 (0.003-0.041)	0.137 (0.078-0.257)	A: 0.6884; B: 0.6813
Lachnospiraceae	0.167 (0.101-0.237)	0.143 (0.118-0.219)	A: 0.6884; B: 0.6813
Ruminococcaceae	0.032 (0.004-0.116)	0.029 (0.002-0.125)	A: 0.8870; B: 0.9970
Coriobacteriaceae	0.00003 (0.00000-0.00358)	0.00000 (0.00000-0.00006)	A: 0.6884; B: 0.6813
Veillonellaceae	0.017 (0.003-0.122)	0.119 (0.010-0.165)	A: 0.6884; B: 0.6813
Enterobacteriaceae	0.080 (0.016-0.202)	0.129 (0.050-0.192)	A: 0.6884; B: 0.6813
	**Visit 12 months**		
	**Outgrowth of CMA**	**No outgrowth of CMA**	**adj. p-value**
Bifidobacteriaceae	0.052 (0.018-0.143)	0.037 (0.007-0.093)	A: 0.9059; B: 0.8378
Bacteroidaceae	0.114 (0.019-0.258)	0.252 (0.133-0.375)	A: 0.4883; B: 0.5029
Lachnospiraceae	0.194 (0.120-0.242)	0.206 (0.169-0.265)	A: 0.4883; B: 0.5558
Ruminococcaceae	0.040 (0.014-0.217)	0.022 (0.002-0.131)	A: 0.4883; B: 0.5029
Coriobacteriaceae	0.00005 (0.00002-0.02495)	0.00003 (0.00000-0.00596)	A: 0.4883; B: 0.5558
Veillonellaceae	0.051 (0.012-0.159)	0.114 (0.026-0.191)	A: 0.4883; B: 0.5558
Enterobacteriaceae	0.050 (0.012-0.108)	0.016 (0.008-0.046)	A: 0.6889; B: 0.8378

Table S6. Difference in 16S rRNA gene based taxa at family level between allergy groups within visits as determined by Linear Mixed Model (LMM) analysis with outgrowth of CMA, visit and outgrowth of CMA x visit as fixed effects and subject as random effect. Abbreviation: p-adj.: adjusted p-value (p-value corrected for multiple testing using the Benjamini-Hochberg correction). Top 10 features ordered by unadjusted p-value.

**Baseline visit – outgrowth vs no outgrowth of CMA**			
**Family**	**p-value LMM**	**p-adj. LMM**	**Found by LEfSe**
Lachnospiraceae	0.004	0.250	yes
Carnobacteriaceae	0.025	0.487	yes
Atopobiaceae	0.035	0.487	
Enterococcaceae	0.037	0.487	yes
Saccharimonadaceae	0.040	0.487	
Enterobacterales_unclassified	0.046	0.487	
Leuconostocaceae	0.058	0.526	yes
Staphylococcaceae	0.071	0.563	
Streptococcaceae	0.091	0.640	
Clostridia_UCG.014_fa	0.128	0.806	
**Visit 6 months – outgrowth vs no outgrowth of CMA**			
**Family**	**p-value LMM**	**p-adj. LMM**	**Found by LEfSe**
Aerococcaceae	0.004	0.254	yes
Pasteurellaceae	0.008	0.257	yes
Eubacteriaceae	0.058	0.688	yes
Prevotellaceae	0.063	0.688	
Monoglobaceae	0.104	0.688	
Tannerellaceae	0.138	0.688	
Oscillospiraceae	0.152	0.688	
Oscillospirales_fa	0.155	0.688	
Bacteria_unclassified	0.163	0.688	
Muribaculaceae	0.163	0.688	
**Visit 12 months – outgrowth vs no outgrowth of CMA**			
**Family**	**p-value LMM**	**p-adj. LMM**	**Found by LEfSe**
Rikenellaceae	0.005	0.213	yes
Prevotellaceae	0.007	0.213	
Comamonadaceae	0.022	0.453	
Actinomycetaceae	0.036	0.564	yes
Gastranaerophilales_fa	0.079	0.582	
Bacteroidaceae	0.082	0.582	yes
Bacteria_unclassified	0.102	0.582	
Muribaculaceae	0.102	0.582	
RF39_fa	0.102	0.582	
Defluviitaleaceae	0.102	0.582	

Table S7. Significance of difference in 16S rRNA gene based core taxa between visits within each allergy group as determined by Linear Mixed Model (LMM) analysis with Benjamini-Hochberg correction. Upper part of the table: infants that outgrew their CMA at 12 months; lower part of the table: infants that did not outgrew their CMA at 12 months. Abbreviations: Med.(IQR): median relative abundance and interquartile range; 0M: 0 months (baseline); 6M: 6 months; 12M: 12 months; p-adj.: adjusted p-value (p-value corrected for multiple testing using the Benjamini-Hochberg correction). A: model without age as fixed effect, B: model including age as fixed effect.

**Infants that outgrew their CMA at visit 12 months**						
	**Med.(IQR) 0M**	**Med.(IQR) 6M**	**Med.(IQR) 12M**	**p-adj. 0M vs 6M**	**p-adj. 0M vs 12M**	**p-adj. 6M vs 12M**
Bifidobacteriaceae	0.025 (0.001-0.079)	0.059 (0.011-0.195)	0.052 (0.018-0.143)	A:0.8512 B:0.9911	A:0.9395 B:0.9882	A:0.9450 B:0.9943
Bacteroidaceae	0.004 (0.000-0.166)	0.084 (0.003-0.041)	0.114 (0.019-0.258)	A:0.6587 B:0.9911	A:0.1561 B:0.9882	A:0.9146 B:0.9943
Lachnospiraceae	0.116 (0.058-0.198)	0.167 (0.101-0.237)	0.194 (0.120-0.242)	A:0.6587 B:0.9911	A:0.6758 B:0.9882	A:0.9450 B:0.9943
Ruminococcaceae	0.004 (0.000-0.070)	0.032 (0.004-0.116)	0.040 (0.014-0.217)	A:0.3301 B:0.9911	**A:0.0182** B:0.9882	A:0.9146 B:0.9943
Coriobacteriaceae	0.00003 (0.00000-0.00406)	0.00003 (0.00000-0.00358)	0.00005 (0.00002-0.02495)	A:0.9095 B:0.9911	A:0.6758 B:0.9882	A:0.7840 B:0.9943
Veillonellaceae	0.023 (0.002-0.154)	0.017 (0.003-0.122)	0.051 (0.012-0.159)	A:0.8818 B:0.9911	A:0.9847 B:0.9882	A:0.9146 B:0.9943
Enterobacteriaceae	0.218 (0.084-0.350)	0.080 (0.016-0.202)	0.050 (0.012-0.108)	**A:0.0406** B:0.9911	**A:0.00003** B:0.9882	A:0.7840 B:0.9943
**Infants that did not outgrew their CMA at visit 12 months**						
	**Med.(IQR) 0M**	**Med.(IQR) 6M**	**Med.(IQR) 12M**	**p-adj. 0M vs 6M**	**p-adj. 0M vs 12M**	**p-adj. 6M vs 12M**
Bifidobacteriaceae	0.004 (0.001-0.076)	0.087 (0.018-0.143)	0.037 (0.007-0.093)	A:0.4933 B:0.9414	A:0.7403 B:0.9965	A:0.9859 B:0.9997
Bacteroidaceae	0.005 (0.000-0.081)	0.137 (0.078-0.257)	0.252 (0.133-0.375)	A:0.1638 B:0.9414	**A:0.0007** B:0.9965	A:0.8307 B:0.9997
Lachnospiraceae	0.212 (0.165-0.306)	0.143 (0.118-0.219)	0.206 (0.169-0.265)	A:0.6960 B:0.9414	A:0.7403 B:0.9965	A:0.9859 B:0.9997
Ruminococcaceae	0.004 (0.000-0.044)	0.029 (0.002-0.125)	0.022 (0.002-0.131)	A:0.3861 B:0.9414	A:0.7403 B:0.9965	A:0.9859 B:0.9997
Coriobacteriaceae	0.00000 (0.00000-0.00022)	0.00000 (0.00000-0.00006)	0.00003 (0.00000-0.00596)	A:0.7983 B:0.9414	A:0.7403 B:0.9965	A:0.8307 B:0.9997
Veillonellaceae	0.026 (0.006-0.128)	0.119 (0.010-0.165)	0.114 (0.026-0.191)	A:0.6960 B:0.9414	A:0.7403 B:0.9965	A:0.9859 B:0.9997
Enterobacteriaceae	0.165 (0.074-0.373)	0.129 (0.050-0.192)	0.016 (0.008-0.046)	A:0.6960 B:0.9414	**A:0.0007** B:0.9965	A:0.0602 B:0.9997

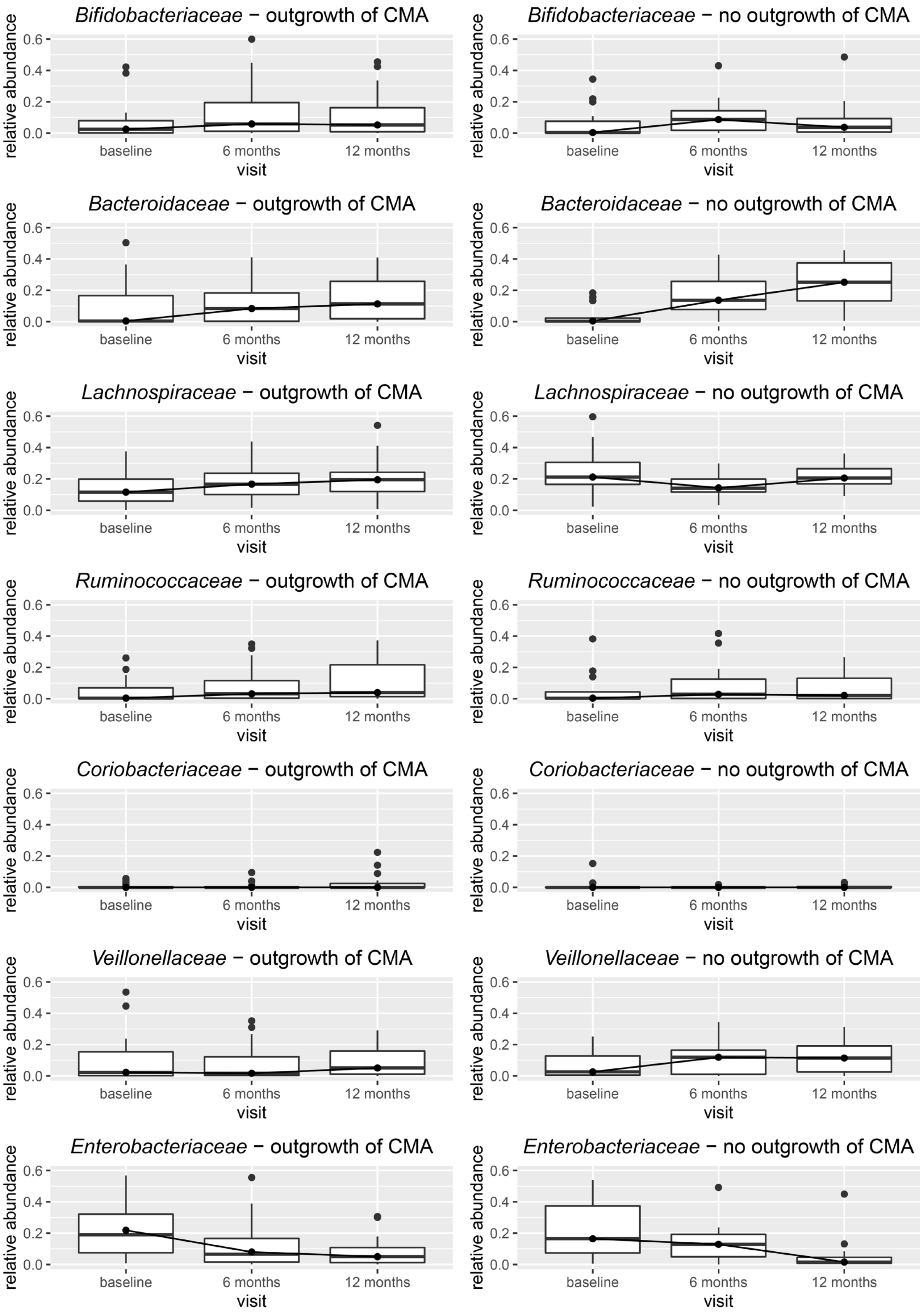

Figure S4. Boxplots of 16S rRNA gene-based relative abundances of core taxa over time.

Table S8. Difference in 16S rRNA gene based taxa at family level between visits within the group which outgrew their CMA as determined by Linear Mixed Model (LMM) analysis with outgrowth of CMA, visit and outgrowth of CMA x visit as fixed effects and subject as random effect. Abbreviation: p-adj.: adjusted p-value (p-value corrected for multiple testing using the Benjamini-Hochberg correction). Significant features ordered by adjusted p-value. In case of less than 10 significant features, the top 10 ordered by unadjusted p-value is presented. Bold = significant after multiple testing correction.

**Outgrowth of CMA – visit 6 months vs baseline**			
**Family**	**p-value LMM**	**p-adj. LMM**	**Found by LEfSe**
Muribaculaceae	0.0001	**0.0018**	
RF39_fa	0.0001	**0.0018**	
Defluviitaleaceae	0.0001	**0.0018**	
Atopobiaceae	0.0004	**0.0045**	
Clostridia_unclassified	0.0005	**0.0045**	
Staphylococcaceae	0.0017	**0.0130**	yes
Micrococcaceae	0.0022	**0.0156**	
Oscillospiraceae	0.0034	**0.0215**	yes
Enterobacterales_unclassified	0.0053	**0.0301**	
Enterobacteriaceae	0.0058	**0.0301**	yes
Eubacteriaceae	0.0062	**0.0301**	yes
**Outgrowth of CMA – visit 12 months vs baseline**			
**Family**	**p-value LMM**	**p-adj. LMM**	**Found by LEfSe**
Muribaculaceae	2.57E-10	**3.24E-09**	
RF39_fa	2.57E-10	**3.24E-09**	
Defluviitaleaceae	2.57E-10	**3.24E-09**	
Clostridia_unclassified	3.73E-07	**3.92E-06**	
Comamonadaceae	6.11E-07	**5.50E-06**	
Enterobacteriaceae	4.79E-06	**3.65E-05**	yes
Atopobiaceae	5.22E-06	**3.65E-05**	
Micrococcaceae	9.60E-06	**6.04E-05**	yes
Enterobacterales_unclassified	1.14E-05	**6.52E-05**	yes
Rikenellaceae	0.0001	**0.0008**	yes
Neisseriaceae	0.0002	**0.0010**	
Staphylococcaceae	0.0013	**0.0056**	
Ruminococcaceae	0.0052	**0.0219**	yes
Enterococcaceae	0.0068	**0.0269**	
Butyricicoccaceae	0.0096	**0.0354**	yes
Oscillospiraceae	0.0110	**0.0385**	yes
Oscillospirales_fa	0.0120	**0.0397**	yes
**Outgrowth of CMA – visit 12 months vs 6 months**			
**Family**	**p-value LMM**	**p-adj. LMM**	**Found by LEfSe**
Comamonadaceae	0.0032	0.0642	
Muribaculaceae	0.0061	0.0642	
RF39_fa	0.0061	0.0642	
Defluviitaleaceae	0.0061	0.0642	
Aerococcaceae	0.0079	0.0714	
Clostridia_UCG.014_fa	0.0249	0.1887	yes
Rikenellaceae	0.0270	0.1887	yes
Neisseriaceae	0.0463	0.2916	
Enterobacteriaceae	0.1138	0.6518	
Clostridia_unclassified	0.1482	0.7779	

Table S9. Difference in 16S rRNA gene based taxa at family level between visits within the group which did not outgrew their CMA as determined by Linear Mixed Model (LMM) analysis with outgrowth of CMA, visit and outgrowth of CMA x visit as fixed effects and subject as random effect. Abbreviation: p-adj.: adjusted p-value (p-value corrected for multiple testing using the Benjamini-Hochberg correction). Significant features ordered by adjusted p-value. In case of less than 10 significant features, the top 10 ordered by unadjusted p-value is presented. Bold = significant after multiple testing correction.

**No outgrowth of CMA – visit 6 months vs baseline**			
**Family**	**p-value LMM**	**p-adj. LMM**	**Found by LEfSe**
Butyricicoccaceae	0.0104	0.6522	yes
Bacteroidaceae	0.0234	0.6946	yes
Actinomycetaceae	0.0331	0.6946	yes
Acidaminococcaceae	0.0557	0.8766	
Ruminococcaceae	0.1103	0.9997	
Clostridiaceae	0.1333	0.9997	
Pasteurellaceae	0.1434	0.9997	
Morganellaceae	0.1500	0.9997	
Peptostreptococcales.Tissierellales_fa	0.1662	0.9997	
Bifidobacteriaceae	0.2114	0.9997	
**No outgrowth of CMA – visit 12 months vs baseline**			
**Family**	**p-value LMM**	**p-adj. LMM**	**Found by LEfSe**
Enterobacteriaceae	0.0002	**0.0066**	yes
Bacteroidaceae	0.0002	**0.0066**	yes
Muribaculaceae	0.0091	0.0816	
RF39_fa	0.0091	0.0816	
Defluviitaleaceae	0.0091	0.0816	
Butyricicoccaceae	0.0122	0.0959	yes
Prevotellaceae	0.0180	0.1258	yes
Actinomycetaceae	0.0233	0.1465	
Micrococcaceae	0.0405	0.2318	
Carnobacteriaceae	0.0488	0.2563	
**No outgrowth of CMA – visit 12 months vs 6 months**			
**Family**	**p-value LMM**	**p-adj. LMM**	**Found by LEfSe**
Enterobacteriaceae	0.0086	0.5393	yes
Monoglobaceae	0.0707	0.6486	yes
Prevotellaceae	0.0790	0.6486	yes
Muribaculaceae	0.0824	0.6486	
RF39_fa	0.0824	0.6486	
Defluviitaleaceae	0.0824	0.6486	
Selenomonadaceae	0.1039	0.7273	yes
Oscillospirales_fa	0.2099	0.9996	yes
Fusobacteriaceae	0.2561	0.9996	
Bacteroidaceae	0.2906	0.9996	

Table S10. Akaike Information Criterion (AIC) values for Linear Mixed Models (LMM) fitted to protein-based based relative abundance of core families. Model A: model without age as fixed effect, model B: model including age as fixed effect. For each core family, the best model (lowest AIC) is indicated in bold.

	**AIC – model A**	**AIC – model B**
Bifidobacteriaceae	**424.9814**	429.6942
Bacteroidaceae	**325.5471**	332.1521
Lachnospiraceae	**307.7449**	314.8004
Ruminococcaceae	**329.2437**	366.3991
Coriobacteriaceae	**503.6151**	508.5317
Veillonellaceae	**524.2340**	529.1699
Enterobacteriaceae	**424.9663**	430.4048

Table S11. Median protein-based relative abundance and interquartile range (IQR) of core families in faeces samples at baseline and follow-up visits (6 months, 12 months) in infants that outgrew their CMA after 12 months (12M) and children that did not. adj. p-value: p-value determined by Linear Mixed Model (LMM) analysis with Benjamini-Hochberg correction for multiple testing. Core taxa were defined as taxa that have a relative abundance higher than 1% in at least 50% of the 16S rRNA gene or metaproteomics samples. A: model without age as fixed effect, B: model including age as fixed effect.

	**Baseline visit**		
	**Outgrowth of CMA**	**No outgrowth of CMA**	**adj. p-value**
Bifidobacteriaceae	0.346 (0.186-0.543)	0.363 (0.127-0.624)	A:0.4304; B:0.4623
Bacteroidaceae	0.024 (0.014-0.047)	0.028 (0.016-0.064)	A:0.4558; B:0.5069
Lachnospiraceae	0.342 (0.256-0.450)	0.441 (0.188-0.597)	A:0.4304; B:0.4623
Ruminococcaceae	0.029 (0.022-0.049)	0.022 (0.020-0.035)	A:0.2125; B:0.2497
Coriobacteriaceae	0.025 (0.001-0.084)	0.048 (0.013-0.096)	A:0.2839; B:0.3398
Veillonellaceae	0.002 (0.001-0.005)	0.001 (0.000-0.003)	A:0.2832; B:0.2497
Enterobacteriaceae	0.012 (0.005-0.038)	0.011 (0.005-0.021)	A:0.2125; B:0.2497
	**Visit 6 months**		
	**Outgrowth of CMA**	**No outgrowth of CMA**	**adj. p-value**
Bifidobacteriaceae	0.359 (0.162-0.692)	0.349 (0.159-0.639)	A:0.5676; B:0.6646
Bacteroidaceae	0.024 (0.017-0.043)	0.028 (0.021-0.041)	A:0.4572; B:0.5095
Lachnospiraceae	0.421 (0.140-0.508)	0.369 (0.236-0.550)	A:0.7562; B:0.7844
Ruminococcaceae	0.037 (0.019-0.076)	0.027 (0.023-0.038)	A:0.3156; B:0.3371
Coriobacteriaceae	0.026 (0.004-0.103)	0.025 (0.013-0.079)	A:0.4311; B:0.4937
Veillonellaceae	0.001 (0.000-0.003)	0.003 (0.002-0.007)	A:0.3156; B:0.3371
Enterobacteriaceae	0.007 (0.004-0.010)	0.005 (0.002-0.014)	A:0.4132; B:0.4870
	**Visit 12 months**		
	**Outgrowth of CMA**	**No outgrowth of CMA**	**adj. p-value**
Bifidobacteriaceae	0.301 (0.133-0.658)	0.347 (0.130-0.460)	A:0.6735; B:0.7277
Bacteroidaceae	0.029 (0.018-0.047)	0.032 (0.016-0.054)	A:0.6735; B:0.7277
Lachnospiraceae	0.298 (0.131-0.517)	0.347 (0.315-0.493)	A:0.6735; B:0.7277
Ruminococcaceae	0.042 (0.013-0.088)	0.035 (0.018-0.056)	A:0.6735; B:0.7277
Coriobacteriaceae	0.124 (0.041-0.181)	0.105 (0.065-0.194)	A:0.6735; B:0.7277
Veillonellaceae	0.002 (0.001-0.004)	0.002 (0.001-0.003)	A:0.6735; B:0.7277
Enterobacteriaceae	0.004 (0.002-0.009)	0.003 (0.003-0.006)	A:0.6735; B:0.7277

Table S12. Difference in protein-based taxa at family level between allergy groups within visits as determined by Linear Mixed Model (LMM) analysis with outgrowth of CMA, visit and outgrowth of CMA x visit as fixed effects and subject as random effect. Abbreviation: p-adj.: adjusted p-value (p-value corrected for multiple testing using the Benjamini-Hochberg correction). Top 10 features ordered by unadjusted p-value.

**Baseline visit – outgrowth vs no outgrowth of CMA**			
**Family**	**p-value LMM**	**p-adj. LMM**	**Found by LEfSe**
Eggerthellaceae	0.009	0.178	yes
Ruminococcaceae	0.049	0.288	
Prevotellaceae	0.056	0.288	
Enterobacteriaceae	0.061	0.288	
Veillonellaceae	0.121	0.393	
Clostridiaceae	0.124	0.393	
Coriobacteriaceae	0.162	0.440	
Erysipelatoclostridiaceae	0.194	0.460	
Bifidobacteriaceae	0.308	0.651	
Lachnospiraceae	0.369	0.701	
**Visit 6 months – outgrowth vs no outgrowth of CMA**			
**Family**	**p-value LMM**	**p-adj. LMM**	**Found by LEfSe**
Prevotellaceae	0.011	0.209	
Veillonellaceae	0.071	0.571	yes
Ruminococcaceae	0.090	0.571	
Enterobacteriaceae	0.177	0.715	
Tannerellaceae	0.243	0.715	
Coriobacteriaceae	0.246	0.715	
Bacteroidaceae	0.327	0.715	
Rikenellaceae	0.359	0.715	
Erysipelatoclostridiaceae	0.370	0.715	
Eggerthellaceae	0.417	0.715	
**Visit 12 months – outgrowth vs no outgrowth of CMA**			
**Family**	**p-value LMM**	**p-adj. LMM**	**Found by LEfSe**
Oscillospiraceae	0.125	0.853	
Lactobacillaceae	0.220	0.853	
Tannerellaceae	0.379	0.853	
Enterobacteriaceae	0.385	0.853	
Prevotellaceae	0.487	0.853	
Erysipelatoclostridiaceae	0.497	0.853	
Clostridiaceae	0.508	0.853	
Eggerthellaceae	0.508	0.853	
Coriobacteriaceae	0.533	0.853	
Bifidobacteriaceae	0.545	0.853	

Table S13. Significance of difference in protein-based core taxa between visits within each allergy group as determined by Linear Mixed Model (LMM) analysis with Benjamini-Hochberg correction. Upper part of the table: infants that outgrew their CMA at 12 months; lower part of the table: infants that did not outgrew their CMA at 12 months. Abbreviations: Med.(IQR): median relative abundance and interquartile range; 0M: 0 months (baseline); 6M: 6 months; 12M: 12 months; p-adj.: adjusted p-value (p-value corrected for multiple testing using the Benjamini-Hochberg correction). A: model without age as fixed effect, B: model including age as fixed effect.

**Infants that outgrew their CMA at visit 12 months**						
	**Med.(IQR) 0M**	**Med.(IQR) 6M**	**Med.(IQR) 12M**	**p-adj. 0M vs 6M**	**p-adj. 0M vs 12M**	**p-adj. 6M vs 12M**
Bifidobacteriaceae	0.346 (0.186-0.543)	0.359 (0.162-0.692)	0.301 (0.133-0.658)	A:0.9649 B:0.9936	A:0.3228 B:0.9786	A:0.7998 B:0.9977
Bacteroidaceae	0.024 (0.014-0.047)	0.024 (0.017-0.043)	0.029 (0.018-0.047)	A:0.9649 B:0.9936	A:0.3228 B:0.9786	A:0.8224 B:0.9977
Lachnospiraceae	0.342 (0.256-0.450)	0.421 (0.140-0.508)	0.298 (0.131-0.517)	A:0.9649 B:0.9936	A:0.1488 B:0.9786	A:0.6659 B:0.9977
Ruminococcaceae	0.029 (0.022-0.049)	0.037 (0.019-0.076)	0.042 (0.013-0.088)	A:0.9649 B:0.9936	A:0.3228 B:0.9786	A:0.6659 B:0.9977
Coriobacteriaceae	0.025 (0.001-0.084)	0.026 (0.004-0.103)	0.124 (0.041-0.181)	A:0.9649 B:0.9936	A:0.1232 B:0.9786	A:0.1184 B:0.9977
Veillonellaceae	0.002 (0.001-0.005)	0.001 (0.000-0.003)	0.002 (0.001-0.004)	A:0.9649 B:0.9936	A:0.8621 B:0.9786	A:0.6659 B:0.9977
Enterobacteriaceae	0.012 (0.005-0.038)	0.007 (0.004-0.010)	0.004 (0.002-0.009)	A:0.9649 B:0.9936	**A:0.0141** B:0.9786	A:0.6659 B:0.9977
**Infants that did not outgrew their CMA at visit 12 months**						
	**Med.(IQR) 0M**	**Med.(IQR) 6M**	**Med.(IQR) 12M**	**p-adj. 0M vs 6M**	**p-adj. 0M vs 12M**	**p-adj. 6M vs 12M**
Bifidobacteriaceae	0.363 (0.127-0.624)	0.349 (0.159-0.639)	0.347 (0.130-0.460)	A:0.9985 B:0.9987	A:0.8975 B:0.9980	A:0.9999 B:0.9994
Bacteroidaceae	0.028 (0.016-0.064)	0.028 (0.021-0.041)	0.032 (0.016-0.054)	A:0.9985 B:0.9987	A:0.8975 B:0.9980	A:0.9999 B:0.9994
Lachnospiraceae	0.441 (0.188-0.597)	0.369 (0.236-0.550)	0.347 (0.315-0.493)	A:0.9985 B:0.9987	A:0.9991 B:0.9980	A:0.9999 B:0.9994
Ruminococcaceae	0.022 (0.020-0.035)	0.027 (0.023-0.038)	0.035 (0.018-0.056)	A:0.9985 B:0.9987	A:0.9991 B:0.9980	A:0.9999 B:0.9994
Coriobacteriaceae	0.048 (0.013-0.096)	0.025 (0.013-0.079)	0.105 (0.065-0.194)	A:0.9985 B:0.9987	A:0.8975 B:0.9980	A:0.9999 B:0.9994
Veillonellaceae	0.001 (0.000-0.003)	0.003 (0.002-0.007)	0.002 (0.001-0.003)	A:0.2456 B:0.9812	A:0.8975 B:0.9980	A:0.9999 B:0.9994
Enterobacteriaceae	0.011 (0.005-0.021)	0.005 (0.002-0.014)	0.003 (0.003-0.006)	A:0.9985 B:0.9987	A:0.8975 B:0.9980	A:0.9999 B:0.9994

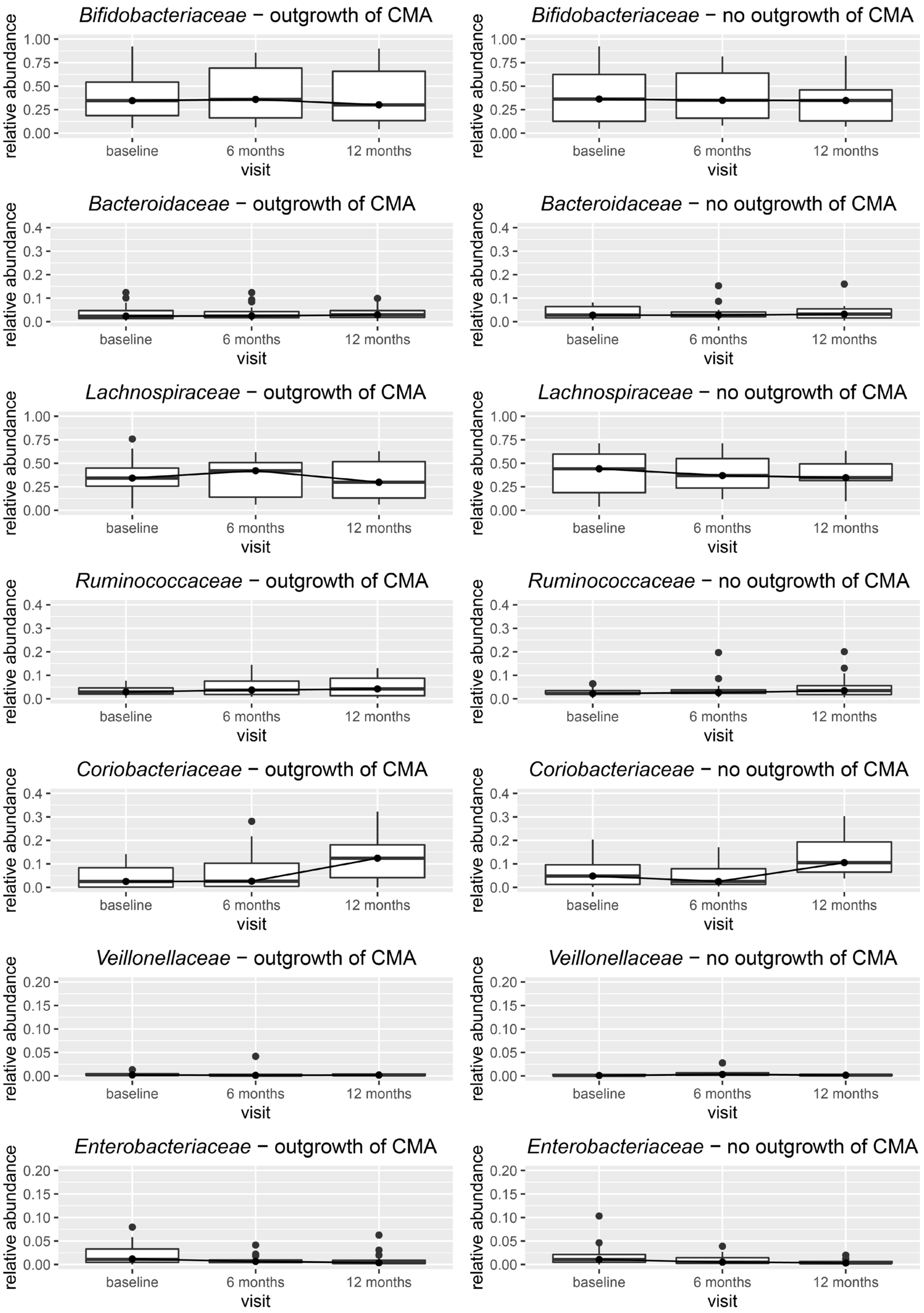

Figure S5. Boxplots of protein-based relative abundances of core taxa over time.

Table S14. Difference in protein-based taxa at family level between visits within the group which outgrew their CMA as determined by Linear Mixed Model (LMM) analysis with outgrowth of CMA, visit and outgrowth of CMA x visit as fixed effects and subject as random effect. Abbreviation: p-adj.: adjusted p-value (p-value corrected for multiple testing using the Benjamini-Hochberg correction). Top 10 features ordered by unadjusted p-value. Bold = significant after multiple testing correction.

**Outgrowth of CMA – visit 6 months vs baseline**			
**Family**	**p-value LMM**	**p-adj. LMM**	**Found by LEfSe**
Eggerthellaceae	0.025	0.479	
Enterobacteriaceae	0.145	0.992	
Prevotellaceae	0.277	0.992	
Acidaminococcaceae	0.319	0.992	
Enterococcaceae	0.339	0.992	
Rikenellaceae	0.516	0.992	
Lachnospiraceae	0.552	0.992	
Bacteroidaceae	0.591	0.992	
Erysipelatoclostridiaceae	0.606	0.992	
Bifidobacteriaceae	0.642	0.992	
**Outgrowth of CMA – visit 12 months vs baseline**			
**Family**	**p-value LMM**	**p-adj. LMM**	**Found by LEfSe**
Enterobacteriaceae	0.002	**0.037**	yes
Enterococcaceae	0.004	**0.037**	
Coriobacteriaceae	0.035	0.223	yes
Lachnospiraceae	0.064	0.275	
Rikenellaceae	0.072	0.275	yes
Eggerthellaceae	0.100	0.318	
Bifidobacteriaceae	0.201	0.545	
Bacteroidaceae	0.262	0.584	
Ruminococcaceae	0.277	0.584	
Tannerellaceae	0.629	0.998	
**Outgrowth of CMA – visit 12 months vs 6 months**			
**Family**	**p-value LMM**	**p-adj. LMM**	**Found by LEfSe**
Coriobacteriaceae	0.017	0.321	yes
Prevotellaceae	0.089	0.842	yes
Enterococcaceae	0.142	0.902	
Enterobacteriaceae	0.238	1.000	
Tannerellaceae	0.301	1.000	
Ruminococcaceae	0.406	1.000	
Lachnospiraceae	0.431	1.000	
Veillonellaceae	0.476	1.000	
Rikenellaceae	0.498	1.000	
Acidaminococcaceae	0.533	1.000	

Table S15. Difference in protein-based taxa at family level between visits within the group which did not outgrew their CMA as determined by Linear Mixed Model (LMM) analysis with outgrowth of CMA, visit and outgrowth of CMA x visit as fixed effects and subject as random effect. Abbreviation: p-adj.: adjusted p-value (p-value corrected for multiple testing using the Benjamini-Hochberg correction). Top 10 features ordered by unadjusted p-value.

**No outgrowth of CMA – visit 6 months vs baseline**			
**Family**	**p-value LMM**	**p-adj. LMM**	**Found by LEfSe**
Veillonellaceae	0.035	0.667	yes
Clostridiaceae	0.195	1.000	yes
Rikenellaceae	0.238	1.000	
Tannerellaceae	0.510	1.000	
Enterococcaceae	0.543	1.000	
Akkermansiaceae	0.572	1.000	
Bacteroidaceae	0.576	1.000	
Enterobacteriaceae	0.594	1.000	
Oscillospiraceae	0.626	1.000	
Acidaminococcaceae	0.651	1.000	
**No outgrowth of CMA – visit 12 months vs baseline**			
**Family**	**p-value LMM**	**p-adj. LMM**	**Found by LEfSe**
Enterococcaceae	0.055	0.796	yes
Prevotellaceae	0.098	0.796	
Tannerellaceae	0.129	0.796	
Enterobacteriaceae	0.181	0.796	yes
Rikenellaceae	0.210	0.796	
Oscillospiraceae	0.313	0.805	
Eggerthellaceae	0.334	0.805	
Veillonellaceae	0.413	0.805	
Coriobacteriaceae	0.416	0.805	yes
Lactobacillaceae	0.424	0.805	
**No outgrowth of CMA – visit 12 months vs 6 months**			
**Family**	**p-value LMM**	**p-adj. LMM**	**Found by LEfSe**
Eggerthellaceae	0.166	1.000	yes
Coriobacteriaceae	0.204	1.000	yes
Prevotellaceae	0.291	1.000	
Enterococcaceae	0.403	1.000	
Veillonellaceae	0.422	1.000	yes
Lactobacillaceae	0.437	1.000	
Tannerellaceae	0.672	1.000	
Clostridiaceae	0.684	1.000	
Enterobacteriaceae	0.697	1.000	
Erysipelatoclostridiaceae	0.770	1.000	

Table S16. Akaike Information Criterion (AIC) values for Linear Mixed Models (LMM) fitted to top 10 microbial protein functional classes (KEGG Brite hierarchy level c). Model A: model without age as fixed effect, model B: model including age as fixed effect. For each functional class, the best model (lowest AIC) is indicated in bold.

	**AIC – model A**	**AIC – model B**
Ribosome	**389.7372**	393.1967
Oxidative phosphorylation	**512.4582**	514.2544
ABC transporters	**403.3784**	409.0291
Glycolysis / Gluconeogenesis	**261.7322**	266.7850
Purine metabolism	**382.7607**	388.2610
RNA degradation	**313.6898**	314.2276
Aminoacyl tRNA biosynthesis	**483.9525**	487.8248
Fructose and mannose metabolism	**460.9811**	464.6671
Pyruvate metabolism	**321.6124**	328.6667
Pentose and glucuronate interconversions	569.1572	**567.4152**

Table S17. Median relative abundance and interquartile range (IQR) of top 10 microbial protein functional classes (KEGG Brite hierarchy level c) in faeces samples at baseline and follow-up visits (6 months, 12 months) in infants that outgrew their CMA after 12 months (12M) and children that did not. adj. p-value: p-value determined by Linear Mixed Model (LMM) analysis with Benjamini-Hochberg correction for multiple testing. A: model without age as fixed effect, B: model including age as fixed effect.

	**Baseline visit**		
	**Outgrowth of CMA**	**No outgrowth of CMA**	**adj. p-value**
Ribosome	0.042 (0.020-0.115)	0.073 (0.049-0.136)	A:0.5704; B:0.5576
Oxidative phosphorylation	0.001 (0.000-0.003)	0.001 (0.000-0.002)	A:0.9732; B:0.8056
ABC transporters	0.003 (0.001-0.007)	0.005 (0.002-0.009)	A:0.8191; B:0.7399
Glycolysis / Gluconeogenesis	**0.006 (0.003-0.008) Min=0.001, max=0.019**	**0.006 (0.003-0.008) Min=0.001, max = 0.024**	**A:0.0154; B:0.0285**
Purine metabolism	0.003 (0.002-0.006)	0.005 (0.002-0.007)	A:0.5704; B:0.6574
RNA degradation	**0.006 (0.005-0.008)**	**0.004 (0.003-0.006)**	**A:0.0439;** B:0.0867
Aminoacyl tRNA biosynthesis	0.001 (0.000-0.004)	0.003 (0.002-0.006)	A:0.5109; B:0.3949
Fructose and mannose metabolism	0.001 (0.000-0.004)	0.002 (0.001-0.004)	A:0.5704; B:0.7144
Pyruvate metabolism	0.002 (0.001-0.004)	0.004 (0.001-0.007)	A:0.2425; B:0.2654
Pentose and glucuronate interconversions	**0.000 (0.000-0.001)**	**0.000 (0.000-0.002)**	**A:0.0439;** B:0.0867
	**Visit 6 months**		
	**Outgrowth of CMA**	**No outgrowth of CMA**	**adj. p-value**
Ribosome	0.087 (0.053-0.161)	0.103 (0.061-0.156)	A:0.9492; B:0.9427
Oxidative phosphorylation	0.002 (0.001-0.004)	0.002 (0.001-0.003)	A:0.9492; B:0.9427
ABC transporters	0.008 (0.004-0.011)	0.008 (0.003-0.012)	A:0.9492; B:0.9427
Glycolysis / Gluconeogenesis	0.007 (0.004-0.010)	0.007 (0.004-0.008)	A:0.9492; B:0.9427
Purine metabolism	0.005 (0.003-0.007)	0.005 (0.003-0.009)	A:0.9492; B:0.9427
RNA degradation	0.005 (0.003-0.006)	0.004 (0.002-0.007)	A:0.9492; B:0.9427
Aminoacyl tRNA biosynthesis	0.004 (0.001-0.010)	0.003 (0.002-0.008)	A:0.9492; B:0.9427
Fructose and mannose metabolism	0.002 (0.000-0.005)	0.002 (0.001-0.003)	A:0.9492; B:0.9427
Pyruvate metabolism	0.005 (0.002-0.008)	0.004 (0.002-0.006)	A:0.9492; B:0.9427
Pentose and glucuronate interconversions	0.001 (0.000-0.004)	0.002 (0.000-0.003)	A:0.9492; B:0.9427
	**Visit 12 months**		
	**Outgrowth of CMA**	**No outgrowth of CMA**	**adj. p-value**
Ribosome	0.083 (0.058-0.130)	0.096 (0.058-0.150)	A:0.9694; B:0.9862
Oxidative phosphorylation	0.003 (0.001-0.006)	0.003 (0.001-0.007)	A:0.9694; B:0.9862
ABC transporters	0.003 (0.002-0.009)	0.004 (0.003-0.005)	A:0.9694; B:0.9862
Glycolysis / Gluconeogenesis	0.007 (0.005-0.011)	0.005 (0.003-0.009)	A:0.9694; B:0.9862
Purine metabolism	0.004 (0.003-0.006)	0.005 (0.004-0.007)	A:0.9694; B:0.9862
RNA degradation	0.004 (0.002-0.005)	0.003 (0.002-0.004)	A:0.9694; B:0.9862
Aminoacyl tRNA biosynthesis	0.005 (0.002-0.009)	0.005 (0.003-0.006)	A:0.9694; B:0.9862
Fructose and mannose metabolism	0.003 (0.002-0.006)	0.004 (0.002-0.008)	A:0.9694; B:0.9862
Pyruvate metabolism	0.005 (0.003-0.007)	0.003 (0.003-0.005)	A:0.9694; B:0.9862
Pentose and glucuronate interconversions	0.002 (0.001-0.003)	0.002 (0.001-0.002)	A:0.9694; B:0.9862

Table S18. Difference in protein functional classes (KEGG Brite hierarchy level c) between allergy groups within visits as determined by Linear Mixed Model (LMM) analysis with outgrowth of CMA, visit and outgrowth of CMA x visit as fixed effects and subject as random effect. Abbreviation: p-adj.: adjusted p-value (p-value corrected for multiple testing using the Benjamini-Hochberg correction). Top 10 features ordered by unadjusted p-value.

**Baseline visit – outgrowth vs no outgrowth of CMA**			
**Functional class**	**p-value LMM**	**p-adj. LMM**	**Found by LEfSe**
Pentose and glucuronate interconversions	0.006	0.334	yes
Inositol phosphate metabolism	0.029	0.334	yes
Pyrimidine metabolism	0.031	0.334	yes
Glycolysis / Gluconeogenesis	0.032	0.334	
Nicotinate and nicotinamide metabolism	0.033	0.334	
Selenocompound metabolism	0.037	0.334	yes
Glyoxylate and dicarboxylate metabolism	0.039	0.334	
Valine, leucine and isoleucine degradation	0.040	0.334	yes
Histidine metabolism	0.041	0.334	
Streptomycin biosynthesis	0.043	0.334	yes
**Visit 6 months – outgrowth vs no outgrowth of CMA**			
**Functional class**	**p-value LMM**	**p-adj. LMM**	**Found by LEfSe**
Amino sugar and nucleotide sugar metabolism	0.006	0.488	yes
Pyrimidine metabolism	0.013	0.569	yes
Glycine, serine and threonine metabolism	0.023	0.656	yes
Biosynthesis of ansamycins	0.055	0.983	
Lysine biosynthesis	0.106	0.983	
Starch and sucrose metabolism	0.121	0.983	
Glutathione metabolism	0.138	0.983	
Phosphotransferase system	0.214	0.983	
Vitamin B6 metabolism	0.215	0.983	
Pentose and glucuronate interconversions	0.233	0.983	
**Visit 12 months – outgrowth vs no outgrowth of CMA**			
**Functional class**	**p-value LMM**	**p-adj. LMM**	**Found by LEfSe**
Sphingolipid metabolism	0.057	0.998	
Other glycan degradation	0.057	0.998	
Amino sugar and nucleotide sugar metabolism	0.088	0.998	
Lysine biosynthesis	0.095	0.998	
Valine, leucine and isoleucine biosynthesis	0.104	0.998	
Pantothenate and CoA biosynthesis	0.123	0.998	
Methane metabolism	0.130	0.998	
Starch and sucrose metabolism	0.144	0.998	yes
Glutathione metabolism	0.147	0.998	
Biofilm formation	0.193	0.998	

Table S19. Significance of difference in top 10 microbial protein functional classes (KEGG Brite hierarchy level c) between visits within each allergy group as determined by Linear Mixed Model (LMM) analysis with Benjamini-Hochberg correction. Upper part of the table: infants that outgrew their CMA at 12 months; lower part of the table: infants that did not outgrew their CMA at 12 months. Abbreviations: Med.(IQR): median relative abundance and interquartile range; 0M: 0 months (baseline); 6M: 6 months; 12M: 12 months; p-adj.: adjusted p-value (p-value corrected for multiple testing using the Benjamini-Hochberg correction). A: model without age as fixed effect, B: model including age as fixed effect.

**Infants that outgrew their CMA at visit 12 months**						
	**Med.(IQR) 0M**	**Med.(IQR) 6M**	**Med.(IQR) 12M**	**p-adj. 0M vs 6M**	**p-adj. 0M vs 12M**	**p-adj. 6M vs 12M**
Ribosome	0.042 (0.020-0.115)	0.087 (0.053-0.161)	0.083 (0.058-0.130)	A:0.9904 B:0.8351	A:0.9928 B:0.6884	A:0.8819 B:1.0000
Oxidative phosphorylation	0.001 (0.000-0.003)	0.002 (0.001-0.004)	0.003 (0.001-0.006)	A:0.9904 B:0.8351	A:0.2481 B:0.9973	A:0.5793 B:1.0000
ABC transporters	0.003 (0.001-0.007)	0.008 (0.004-0.011)	0.003 (0.002-0.009)	A:0.9904 B:0.9912	A:0.2481 B:0.4802	A:0.1814 B:0.1814
Glycolysis / Gluconeogenesis	0.006 (0.003-0.008)	0.007 (0.004-0.010)	0.007 (0.005-0.011)	**A:0.0413** B:0.0608	**A:0.0039 B:0.0078**	A:0.8819 B:1.0000
Purine metabolism	0.003 (0.002-0.006)	0.005 (0.003-0.007)	0.004 (0.003-0.006)	A:0.9904 B:0.8351	A:0.3657 B: 0.9973	A:0.4538 B:1.0000
RNA degradation	0.006 (0.005-0.008)	0.005 (0.003-0.006)	0.004 (0.002-0.005)	**A:0.0283** B:0.8351	**A:< 10^{-4}** B:0.9973	A:0.2859 B:1.0000
Aminoacyl tRNA biosynthesis	0.001 (0.000-0.004)	0.004 (0.001-0.010)	0.005 (0.002-0.009)	A:0.0892 B:0.0608	A:0.1056 B:0.1458	A:0.9820 B:1.0000
Fructose and mannose metabolism	0.001 (0.000-0.004)	0.002 (0.000-0.005)	0.003 (0.002-0.006)	A:0.9904 B:0.8351	A:0.4031 B:0.9973	A:0.7269 B:1.0000
Pyruvate metabolism	0.002 (0.001-0.004)	0.005 (0.002-0.008)	0.005 (0.003-0.007)	A:0.9904 B:0.8351:	A:0.2760 B:0.9973	A:0.8819 B:1.0000
Pentose and glucuronate interconversions	0.000 (0.000-0.001)	0.001 (0.000-0.004)	0.002 (0.001-0.003)	A:0.9904 B:0.8351	**A:0.0166 B:0.0248**	A:0.1814 B:0.1814
**Infants that did not outgrew their CMA at visit 12 months**						
	**Med.(IQR) 0M**	**Med.(IQR) 6M**	**Med.(IQR) 12M**	**p-adj. 0M vs 6M**	**p-adj. 0M vs 12M**	**p-adj. 6M vs 12M**
Ribosome	0.073 (0.049-0.136)	0.103 (0.061-0.156)	0.096 (0.058-0.150)	A:0.9984 B:0.9946	A:0.9922 B:0.9998	A:0.9736 B:0.9936
Oxidative phosphorylation	0.001 (0.000-0.002)	0.002 (0.001-0.003)	0.003 (0.001-0.007)	A:0.9984 B:0.9946:	A:0.9922 B:0.9998	A:0.9585 B:0.9936
ABC transporters	0.005 (0.002-0.009)	0.008 (0.003-0.012)	0.004 (0.003-0.005)	A:0.9984 B:0.9946:	A:0.9922 B:0.9998:	A:0.9585 B:0.9936
Glycolysis / Gluconeogenesis	0.006 (0.003-0.008)	0.007 (0.004-0.008)	0.005 (0.003-0.009)	A:0.9984 B:0.9946	A:0.9922 B:0.9998	A:0.9585 B:0.9936
Purine metabolism	0.005 (0.002-0.007)	0.005 (0.003-0.009)	0.005 (0.004-0.007)	A:0.9984 B:0.9946	A:0.9922 B:0.9998	A:0.9736 B:0.9936
RNA degradation	0.004 (0.003-0.006)	0.004 (0.002-0.007)	0.003 (0.002-0.004)	A:0.9984 B:0.9946	A:0.6915 B:0.9998	A:0.9585 B:0.9936
Aminoacyl tRNA biosynthesis	0.003 (0.002-0.006)	0.003 (0.002-0.008)	0.005 (0.003-0.006)	A:0.9984 B:0.9946	A:0.9922 B:0.9998	A:0.9736 B:0.9936
Fructose and mannose metabolism	0.002 (0.001-0.004)	0.002 (0.001-0.003)	0.004 (0.002-0.008)	A:0.9984 B:0.9946	A:0.9922 B:0.9998:	A:0.9736 B:0.9936
Pyruvate metabolism	0.004 (0.001-0.007)	0.004 (0.002-0.006)	0.003 (0.003-0.005)	A:0.9984 B:0.9946	A:0.9922 B:0.9998	A:0.9736 B:0.9936
Pentose and glucuronate interconversions	0.000 (0.000-0.002)	0.002 (0.000-0.003)	0.002 (0.001-0.002)	A:0.9984 B:0.9946	A:0.9922 B:0.9998	A:0.9736 B:0.9936

Table S20. Difference in protein functional classes (KEGG Brite hierarchy level c) between visits within the group which outgrew their CMA as determined by Linear Mixed Model (LMM) analysis with outgrowth of CMA, visit and outgrowth of CMA x visit as fixed effects and subject as random effect. Abbreviation: p-adj.: adjusted p-value (p-value corrected for multiple testing using the Benjamini-Hochberg correction). Top 10 features ordered by unadjusted p-value. Bold = significant after multiple testing correction.

**Outgrowth of CMA – visit 6 months vs baseline**			
**Functional class**	**p-value LMM**	**p-adj. LMM**	**Found by LEfSe**
Nicotinate and nicotinamide metabolism	0.0052	0.3578	
Aminoacyl-tRNA biosynthesis	0.0095	0.3578	yes
Selenocompound metabolism	0.0294	0.5017	yes
RNA degradation	0.0295	0.5017	
Two component system	0.0410	0.5291	
Carbon fixation pathways in prokaryotes	0.0469	0.5291	
Cyanoamino acid metabolism	0.0558	0.5291	
Riboflavin metabolism	0.0560	0.5291	
Biofilm formation	0.1104	0.8074	
Galactose metabolism	0.1314	0.8074	yes
**Outgrowth of CMA – visit 12 months vs baseline**			
**Functional class**	**p-value LMM**	**p-adj. LMM**	**Found by LEfSe**
Selenocompound metabolism	0.0001	**0.0104**	yes
RNA degradation	0.0002	**0.0104**	
Tryptophan metabolism	0.0023	**0.0484**	yes
Carbon fixation pathways in prokaryotes	0.0028	**0.0484**	
Propanoate metabolism	0.0038	0.0513	yes
Cyanoamino acid metabolism	0.0044	0.0513	
Pentose and glucuronate interconversions	0.0048	0.0513	yes
Lysine degradation	0.0108	0.0876	
Terpenoid backbone biosynthesis	0.0108	0.0876	
Aminoacyl-tRNA biosynthesis	0.0113	0.0876	yes
**Outgrowth of CMA – visit 12 months vs 6 months**			
**Functional class**	**p-value LMM**	**p-adj. LMM**	**Found by LEfSe**
Flagellar assembly	0.0205	0.8566	yes
Pentose and glucuronate interconversions	0.0449	0.8566	
Amino sugar and nucleotide sugar metabolism	0.0558	0.8566	yes
Sphingolipid metabolism	0.0583	0.8566	
Other glycan degradation	0.0583	0.8566	
Tryptophan metabolism	0.0683	0.8566	
ABC transporters	0.0705	0.8566	
Fatty acid degradation	0.1208	0.9412	
Vitamin B6 metabolism	0.1326	0.9412	
Lysine degradation	0.1455	0.9412	

Table S21. Difference in protein functional classes (KEGG Brite hierarchy level c) between visits within the group which did not outgrew their CMA as determined by Linear Mixed Model (LMM) analysis with outgrowth of CMA, visit and outgrowth of CMA x visit as fixed effects and subject as random effect. Abbreviation: p-adj.: adjusted p-value (p-value corrected for multiple testing using the Benjamini-Hochberg correction). Top 10 features ordered by unadjusted p-value.

**No outgrowth of CMA – visit 6 months vs baseline**			
**Functional class**	**p-value LMM**	**p-adj. LMM**	**Found by LEfSe**
Carbon fixation pathways in prokaryotes	0.0272	0.9997	
Amino sugar and nucleotide sugar metabolism	0.0681	0.9997	
Glutathione metabolism	0.0831	0.9997	yes
Flagellar assembly	0.0936	0.9997	
Taurine and hypotaurine metabolism	0.1871	0.9997	
Lysine degradation	0.2173	0.9997	
Terpenoid backbone biosynthesis	0.2173	0.9997	
Histidine metabolism	0.2588	0.9997	
Starch and sucrose metabolism	0.2655	0.9997	
Tryptophan metabolism	0.2860	0.9997	
**No outgrowth of CMA – visit 12 months vs baseline**			
**Functional class**	**p-value LMM**	**p-adj. LMM**	**Found by LEfSe**
Tryptophan metabolism	0.0054	0.4564	yes
Sphingolipid metabolism	0.0169	0.4798	
Other glycan degradation	0.0169	0.4798	
Carbon fixation pathways in prokaryotes	0.0249	0.5287	
Lysine biosynthesis	0.1680	0.9999	yes
Lysine degradation	0.1766	0.9999	
Terpenoid backbone biosynthesis	0.1766	0.9999	
Cyanoamino acid metabolism	0.2079	0.9999	
RNA degradation	0.2534	0.9999	
Glycine, serine and threonine metabolism	0.2673	0.9999	
**No outgrowth of CMA – visit 12 months vs 6 months**			
**Functional class**	**p-value LMM**	**p-adj. LMM**	**Found by LEfSe**
Flagellar assembly	0.0058	0.2511	yes
Sphingolipid metabolism	0.0089	0.2511	
Other glycan degradation	0.0089	0.2511	
Glutathione metabolism	0.0188	0.3992	yes
Starch and sucrose metabolism	0.0590	0.9999	yes
Amino sugar and nucleotide sugar metabolism	0.0716	0.9999	
Oxidative phosphorylation	0.1352	0.9999	
Biosynthesis of various plant secondary metabolites	0.1765	0.9999	
Tryptophan metabolism	0.2137	0.9999	
Pantothenate and CoA biosynthesis	0.2164	0.9999	yes

Table S22. Akaike Information Criterion (AIC) values for Linear Mixed Models (LMM) fitted to top 10 human protein classes. Model A: model without age as fixed effect, model B: model including age as fixed effect. For each functional class, the best model (lowest AIC) is indicated in bold.

	**AIC – model A**	**AIC – model B**
Immunoglobulins	**375.8744**	376.0734
Glycoside hydrolases	**426.9341**	433.0265
Transthyretin/hydroxyisourate hydrolases	**613.9620**	614.0648
Proline-rich proteins	**605.7402**	609.7291
S100 proteins	**501.4520**	505.8600
Secretory proteins	**540.8112**	544.3607
Serine peptidases	**470.7533**	472.6541
Carboxypeptidases	**387.2018**	393.1329
Dipeptidyl peptidases	**573.0248**	576.8840
Actin family	**528.2970**	530.2747

Table S23. Median relative abundance and interquartile range (IQR) of top 10 human protein classes in faeces samples at baseline and follow-up visits (6 months, 12 months) in infants that outgrew their CMA after 12 months (12M) and children that did not. adj. p-value: p-value determined by Linear Mixed Model (LMM) analysis with Benjamini-Hochberg correction for multiple testing. A: model without age as fixed effect, B: model including age as fixed effect.

	**Baseline visit**		
	**Outgrowth of CMA**	**No outgrowth of CMA**	**adj. p-value**
Immunoglobulins	0.530 (0.324-0.657)	0.645 (0.544-0.770)	A:0.8517; B:0.5278
Glycoside hydrolases	0.009 (0.006-0.031)	0.010 (0.006-0.021)	A:0.9510; B:0.9512
Transthyretin/hydroxyisourate hydrolases	0.013 (0.000-0.030)	0.006 (0.000-0.025)	A:0.9510; B:0.9512
Proline-rich proteins	0.001 (0.000-0.007)	0.001 (0.000-0.008)	A:0.9510; B:0.9512
S100 proteins	0.012 (0.007-0.025)	0.006 (0.002-0.009)	A:0.8517; B:0.5278
Secretory proteins	0.002 (0.002-0.011)	0.003 (0.000-0.016)	A:0.9510; B:0.9512
Serine peptidases	0.167 (0.033-0.384)	0.128 (0.073-0.235)	A:0.9510; B:0.9512
Carboxypeptidases	0.007 (0.001-0.015)	0.004 (0.001-0.013)	A:0.9510; B:0.9512
Dipeptidyl peptidases	0.003 (0.001-0.007)	0.001 (0.000-0.009)	A:0.9510; B:0.9512
Actin family	0.001 (0.000-0.003)	0.001 (0.000-0.002)	A:0.9510; B:0.9512
	**Visit 6 months**		
	**Outgrowth of CMA**	**No outgrowth of CMA**	**adj. p-value**
Immunoglobulins	0.497 (0.295-0.639)	0.593 (0.461-0.746)	A:0.7298; B:0.9071
Glycoside hydrolases	0.014 (0.010-0.048)	0.025 (0.008-0.042)	A:0.7298; B:0.7697
Transthyretin/hydroxyisourate hydrolases	0.002 (0.000-0.022)	0.013 (0.002-0.022)	A:0.4709; B:0.6573
Proline-rich proteins	0.005 (0.001-0.018)	0.008 (0.006-0.019)	A:0.4709; B:0.6573
S100 proteins	0.005 (0.001-0.017)	0.002 (0.001-0.009)	A:0.7298; B:0.7697
Secretory proteins	0.004 (0.002-0.008)	0.010 (0.002-0.021)	A:0.7298; B:0.7697
Serine peptidases	0.213 (0.152-0.424)	0.110 (0.070-0.324)	A:0.3296; B:0.5091
Carboxypeptidases	0.003 (0.002-0.011)	0.007 (0.003-0.011)	A:0.7298; B:0.6798
Dipeptidyl peptidases	0.002 (0.000-0.008)	0.001 (0.000-0.003)	A:0.4709; B:0.5932
Actin family	0.001 (0.000-0.005)	0.004 (0.001-0.010)	A:0.4709; B:0.5932
	**Visit 12 months**		
	**Outgrowth of CMA**	**No outgrowth of CMA**	**adj. p-value**
Immunoglobulins	0.514 (0.366-0.636)	0.570 (0.431-0.672)	A:0.9011; B:0.6762
Glycoside hydrolases	0.032 (0.015-0.045)	0.043 (0.034-0.084)	A:0.4855; B:0.4862
Transthyretin/hydroxyisourate hydrolases	0.016 (0.007-0.024)	0.006 (0.002-0.020)	A:0.4892; B:0.4862
Proline-rich proteins	0.010 (0.004-0.065)	0.028 (0.015-0.046)	A:0.4855; B:0.4862
S100 proteins	0.010 (0.003-0.037)	0.007 (0.002-0.018)	A:0.4855; B:0.4862
Secretory proteins	0.002 (0.001-0.007)	0.003 (0.002-0.006)	A:0.8664; B:0.6762
Serine peptidases	0.160 (0.122-0.379)	0.135 (0.091-0.196)	A:0.4855; B:0.4862
Carboxypeptidases	0.012 (0.004-0.025)	0.017 (0.010-0.028)	A:0.4855; B:0.4862
Dipeptidyl peptidases	0.013 (0.007-0.022)	0.005 (0.001-0.026)	A:0.4855; B:0.4862
Actin family	0.002 (0.001-0.005)	0.002 (0.001-0.014)	A:0.4855; B:0.4862

Table S24. Difference in human protein classes between allergy groups within visits as determined by Linear Mixed Model (LMM) analysis with outgrowth of CMA, visit and outgrowth of CMA x visit as fixed effects and subject as random effect. Abbreviation: p-adj.: adjusted p-value (p-value corrected for multiple testing using the Benjamini-Hochberg correction). Top 10 features ordered by unadjusted p-value.

**Baseline visit – outgrowth vs no outgrowth of CMA**			
**Human protein class**	**p-value LMM**	**p-adj. LMM**	**Found by LEfSe**
Cell adhesion proteins	0.019	0.977	
Mucin family	0.048	0.977	
Superoxide dismutases	0.054	0.977	
F ATPases	0.071	0.977	
Annexins	0.096	0.977	
S100 proteins	0.100	0.977	yes
Elongation factors	0.122	0.977	
Alkaline phosphatases	0.152	0.977	yes
Binding protein	0.158	0.977	
Transmembrane ATPases	0.190	0.977	
**Visit 6 months – outgrowth vs no outgrowth of CMA**			
**Human protein class**	**p-value LMM**	**p-adj. LMM**	**Found by LEfSe**
Peroxidases	0.002	0.148	
Exosomal proteins	0.004	0.177	yes
Actin family	0.066	0.921	yes
Carboxylic ester hydrolases	0.082	0.921	
Lectins	0.098	0.921	
F-ATPases	0.098	0.921	
CUB-domain containing proteins	0.103	0.921	
Titin	0.103	0.921	
Phosphoglycerate kinase	0.110	0.921	
Complement C3 like proteins	0.114	0.921	
**Visit 12 months – outgrowth vs no outgrowth of CMA**			
**Human protein class**	**p-value LMM**	**p-adj. LMM**	**Found by LEfSe**
Serpins	0.004	0.322	yes
Tumor suppressor	0.017	0.803	
Adenosine deaminases	0.064	0.933	
P-ATPases	0.082	0.933	yes
Cadherins	0.086	0.933	
Ubiquitins	0.092	0.933	
Bile salt activated lipase	0.099	0.933	
F-ATPases	0.111	0.933	
Submaxillary gland androgen regulated proteins	0.140	0.933	
CUB-domain containing proteins	0.153	0.933	

Table S25. Significance of difference in top 10 human protein classes between visits within each allergy group as determined by Linear Mixed Model (LMM) analysis with Benjamini-Hochberg correction. Upper part of the table: infants that outgrew their CMA at 12 months; lower part of the table: infants that did not outgrew their CMA at 12 months. Abbreviations: Med.(IQR): median relative abundance and interquartile range; 0M: 0 months (baseline); 6M: 6 months; 12M: 12 months; p-adj.: adjusted p-value (p-value corrected for multiple testing using the Benjamini-Hochberg correction). A: model without age as fixed effect, B: model including age as fixed effect.

**Infants that outgrew their CMA at visit 12 months**						
	**Med.(IQR) 0M**	**Med.(IQR) 6M**	**Med.(IQR) 12M**	**p-adj. 0M vs 6M**	**p-adj. 0M vs 12M**	**p-adj. 6M vs 12M**
Immunoglobulins	0.530 (0.324-0.657)	0.497 (0.295-0.639)	0.514 (0.366-0.636)	A:1.0000 B:0.5077	A:0.0527 B:0.9965	A:0.0764 B:0.9595
Glycoside hydrolases	0.009 (0.006-0.031)	0.014 (0.010-0.048)	0.032 (0.015-0.045)	A:1.0000 B:0.8435	A:0.9863 B:0.9965	A:0.9051 B:0.9595
Transthyretin/hydroxyisourate hydrolases	0.013 (0.000-0.030)	0.002 (0.000-0.022)	0.016 (0.007-0.024)	A:1.0000 B:0.1935	A:0.5965 B:0.9159	A:0.0764 B:0.9595
Proline-rich proteins	0.001 (0.000-0.007)	0.005 (0.001-0.018)	0.010 (0.004-0.065)	A:0.2247 B:0.5965	**A:0.0339** B:0.9159	A:0.9051 B:0.9595
S100 proteins	0.012 (0.007-0.025)	0.005 (0.001-0.017)	0.010 (0.003-0.037)	**A:0.0429** B:0.1103	A:0.0527 B:0.9159	A:0.9398 B:0.9595
Secretory proteins	0.002 (0.002-0.011)	0.004 (0.002-0.008)	0.002 (0.001-0.007)	A:1.0000 B:0.8435	A:0.4763 B:0.9965	A:0.3249 B:0.9595
Serine peptidases	0.167 (0.033-0.384)	0.213 (0.152-0.424)	0.160 (0.122-0.379)	A:1.0000 B:0.2652	A:0.9099 B:0.9159	A:0.3223 B:0.9595
Carboxypeptidases	0.007 (0.001-0.015)	0.003 (0.002-0.011)	0.012 (0.004-0.025)	A:1.0000 B:0.8435	A:0.9099 B:0.9159	A:0.9398 B:0.9595
Dipeptidyl peptidases	0.003 (0.001-0.007)	0.002 (0.000-0.008)	0.013 (0.007-0.022)	A:1.0000 B:0.8435	A:0.4050 B:0.9965	A:0.3249 B:0.9595
Actin family	0.001 (0.000-0.003)	0.001 (0.000-0.005)	0.002 (0.001-0.005)	A:1.0000 B:0.8329	A:0.9099 B:0.9159	A:0.9051 B:0.9595
**Infants that did not outgrew their CMA at visit 12 months**						
	**Med.(IQR) 0M**	**Med.(IQR) 6M**	**Med.(IQR) 12M**	**p-adj. 0M vs 6M**	**p-adj. 0M vs 12M**	**p-adj. 6M vs 12M**
Immunoglobulins	0.645 (0.544-0.770)	0.593 (0.461-0.746)	0.570 (0.431-0.672)	A:0.5769 B:0.9994	**A:0.0222** B:0.9985	A:0.9798 B:0.9994
Glycoside hydrolases	0.010 (0.006-0.021)	0.025 (0.008-0.042)	0.043 (0.034-0.084)	A:0.9968 B:0.9994	A:0.9998 B:0.9985	A:0.9798 B:0.9994
Transthyretin/hydroxyisourate hydrolases	0.006 (0.000-0.025)	0.013 (0.002-0.022)	0.006 (0.002-0.020)	A:0.9968 B:0.6372	A:0.9998 B:0.8230	A:0.9798 B:0.9994
Proline-rich proteins	0.001 (0.000-0.008)	0.008 (0.006-0.019)	0.028 (0.015-0.046)	A:0.5769 B:0.5256	A:0.0562 B:0.9706	A:0.9798 B:0.9994
S100 proteins	0.006 (0.002-0.009)	0.002 (0.001-0.009)	0.007 (0.002-0.018)	A:0.5769 B:0.5256	A:0.4603 B:0.8230	A:0.9798 B:0.9994
Secretory proteins	0.003 (0.000-0.016)	0.010 (0.002-0.021)	0.003 (0.002-0.006)	A:0.5769 B:0.5256	A:0.9998 B:0.9985	A:0.9798 B:0.9994
Serine peptidases	0.128 (0.073-0.235)	0.110 (0.070-0.324)	0.135 (0.091-0.196)	A:0.5769 B:0.9994	A:0.4298 B:0.9985	A:0.9798 B:0.9994
Carboxypeptidases	0.004 (0.001-0.013)	0.007 (0.003-0.011)	0.017 (0.010-0.028)	A:0.5769 B:0.5298	A:0.9998 B:0.9985	A:0.9798 B:0.9994
Dipeptidyl peptidases	0.001 (0.000-0.009)	0.001 (0.000-0.003)	0.005 (0.001-0.026)	A:0.5769 B:0.5256	A:0.9998 B:0.9985	A:0.9798 B:0.9994
Actin family	0.001 (0.000-0.002)	0.004 (0.001-0.010)	0.002 (0.001-0.014)	A:0.5769 B:0.4352	A:0.9896 B:0.8230	A:0.9798 B:0.9994

Table S26. Difference in human protein classes between visits within the group which outgrew their CMA as determined by Linear Mixed Model (LMM) analysis with outgrowth of CMA, visit and outgrowth of CMA x visit as fixed effects and subject as random effect. Abbreviation: p-adj.: adjusted p-value (p-value corrected for multiple testing using the Benjamini-Hochberg correction). Top 10 features ordered by unadjusted p-value.

**Outgrowth of CMA – visit 6 months vs baseline**			
**Human protein class**	**p-value LMM**	**p-adj. LMM**	**Found by LEfSe**
S100 proteins	0.007	0.649	yes
P-ATPases	0.046	1.000	yes
Proline rich proteins	0.070	1.000	
Cell surface glycoproteins	0.071	1.000	yes
Initiation factors	0.093	1.000	
Annexins	0.107	1.000	
Actin binding proteins	0.126	1.000	
Alkaline phosphatases	0.138	1.000	
Transferases	0.153	1.000	yes
Cadherins	0.240	1.000	
**Outgrowth of CMA – visit 12 months vs baseline**			
**Human protein class**	**p-value LMM**	**p-adj. LMM**	**Found by LEfSe**
Alkaline phosphatases	0.001	0.073	yes
Proline rich proteins	0.002	0.073	yes
Superoxide dismutases	0.004	0.133	yes
Ubiquitins	0.008	0.159	yes
Fused gene family	0.009	0.159	yes
Filaggrins	0.013	0.202	yes
Binding protein	0.016	0.205	yes
Ligases	0.020	0.220	
Ferritins	0.022	0.220	yes
Plakins	0.024	0.220	yes
**Outgrowth of CMA – visit 12 months vs 6 months**			
**Human protein class**	**p-value LMM**	**p-adj. LMM**	**Found by LEfSe**
Filaggrins	0.003	0.163	yes
Fused gene family	0.006	0.163	yes
Transthyretin hydroxyisourate hydrolases	0.007	0.163	
Selenium binding proteins	0.007	0.163	
Triosephosphate isomerase	0.027	0.405	yes
Haemoglobins	0.032	0.405	
Phosphorylase enzymes	0.043	0.405	
Initiation factors	0.044	0.405	
Cadherins	0.048	0.405	yes
Fatty acid binding proteins	0.048	0.405	yes

Table S27. Difference in human protein classes between visits within the group which did not outgrew their CMA as determined by Linear Mixed Model (LMM) analysis with outgrowth of CMA, visit and outgrowth of CMA x visit as fixed effects and subject as random effect. Abbreviation: p-adj.: adjusted p-value (p-value corrected for multiple testing using the Benjamini-Hochberg correction). Top 10 features ordered by unadjusted p-value.

**No outgrowth of CMA – visit 6 months vs baseline**			
**Human protein class**	**p-value LMM**	**p-adj. LMM**	**Found by LEfSe**
Exosomal proteins	0.002	0.149	yes
Cell adhesion proteins	0.008	0.376	yes
Filaggrins	0.039	0.825	yes
Proline rich proteins	0.039	0.825	yes
Actin family	0.045	0.825	yes
Complement C3 like proteins	0.093	1.000	
Serpins	0.095	1.000	yes
Selenium binding proteins	0.101	1.000	
Mucin family	0.111	1.000	
Actin binding proteins	0.129	1.000	yes
**No outgrowth of CMA – visit 12 months vs baseline**			
**Human protein class**	**p-value LMM**	**p-adj. LMM**	**Found by LEfSe**
Tumor suppressor	0.003	0.222	
Cell adhesion proteins	0.006	0.222	
Proline rich proteins	0.007	0.222	yes
Serpins	0.035	0.673	
Neutral alkaline ceramidases	0.037	0.673	yes
Immunoglobulins	0.060	0.746	
Macroglobulins	0.075	0.746	
Calreticulin family	0.083	0.746	
Amylases	0.088	0.746	yes
Triosephosphate isomerase	0.088	0.746	yes
**No outgrowth of CMA – visit 12 months vs 6 months**			
**Human protein class**	**p-value LMM**	**p-adj. LMM**	**Found by LEfSe**
Filaggrins	0.001	0.070	yes
Selenium binding proteins	0.004	0.181	
Exosomal proteins	0.007	0.181	yes
Tumor suppressor	0.008	0.181	
Fatty acid binding proteins	0.052	0.947	yes
Pyridine nucleotide disulphide reductases	0.090	0.947	yes
Immunoglobulins	0.096	0.947	
Ferritins	0.108	0.947	
Peroxidases	0.112	0.947	
Alkaline phosphatases	0.113	0.947	

Table S28. Spearman correlation between human (top 10 human protein classes) and microbial (core taxa) proteins. P-values (between brackets) were determined with Monte Carlo permutation (10 000 permutations). P-values below 0.05 are considered significant.

	Bacteroidaceae	***Bifidobacteriaceae***	***Lachnospiraceae***	***Ruminococcaceae***	***Coriobacteriaceae***	***Enterobacteriaceae***	***Veillonellaceae***
Immunoglobulins	0.0253 (0.7871)	0.1636 (0.0737)	-0.0546 (0.557)	-0.1155 (0.2254)	-0.0180 (0.8512)	-0.0303 (0.7390)	0.0384 (0.6768)
Glycoside hydrolases	0.1698 (0.0669)	-0.1131 (0.2216)	0.0878 (0.3479)	0.1419 (0.1256)	**0.2732 (0.0038)**	**-0.2042 (0.0282)**	-0.0711 (0.4448)
Transthyretin/hydroxyisourate hydrolases	**0.1934 (0.0373)**	**-0.1980 (0.0313)**	0.1279 (0.1693)	0.0191 (0.8361)	**0.4603 (< 10^{-4})**	0.0484 (0.6137)	**0.2968 (0.0010)**
Proline-rich proteins	-0.0426 (0.6503)	-0.1585 (0.0873)	**0.1939 (0.0358)**	**0.2161 (0.0167)**	**0.3513 (0.0001)**	**-0.1953 (0.0341)**	0.1472 (0.1134)
S100 proteins	**0.2828 (0.0023)**	-0.1613 (0.084)	0.0496 (0.5894)	0.1080 (0.2491)	0.1783 (0.0525)	0.1789 (0.0544)	0.1258 (0.1714)
Secretory proteins	**0.2584 (0.0054)**	**-0.2712 (0.0046)**	**0.2508 (0.0082)**	0.1477 (0.1094)	0.1593 (0.0839)	0.0482 (0.6052)	0.1251 (0.1771)
Serine peptidases	-0.0981 (0.2916)	-0.0143 (0.8755)	-0.0412 (0.6648)	-0.0019 (0.9828)	-0.1071 (0.2498)	0.0693 (0.4578)	-0.0509 (0.5878)
Carboxypeptidases	**0.1894 (0.0412)**	**-0.2943 (0.0009)**	**0.2541 (0.0068)**	**0.1895 (0.0380)**	**0.4598 (< 10^{-4})**	-0.0830 (0.3678)	**0.2274 (0.0148)**
Dipeptidyl peptidases	**0.2471 (0.0079)**	-0.1664 (0.0671)	0.1328 (0.1544)	**0.2253 (0.0161)**	**0.2869 (0.0015)**	-0.0678 (0.4690)	-0.0237 (0.7903)
Actin family	0.0685 (0.4579)	-0.1057 (0.2529)	0.1154 (0.2175)	**0.1941 (0.0378)**	**0.1939 (0.0354)**	-0.1089 (0.2528)	0.1551 (0.0956)

Table S29. Significant (p-value ≤ 0.05, bold italic) and marginally significant (0.05 < p-value ≤ 0.1) results from redundancy analysis (RDA) on microbial proteome profiles, microbial proteome functional profiles (KEGG Brite level c), 16S rRNA gene-based taxonomic profiles (family level), protein-based taxonomic profiles (family level) and human protein profiles. adj. p-value: p-value adjusted for multiple testing using the Benjamini-Hochberg correction.

**Type of profile**	**Baseline**			**6 months**			**12 months**		
	**Variable**	**adj. p-value**	**%variance explained by selected variables**	**Variable**	**adj. p-value**	**%variance explained by selected variables**	**Variable**	**adj. p-value**	**%variance explained by selected variables**
**Microbial proteome**	none	n/a	n/a	delivery<br>treatment<br>age<br>egg allergy<br>outgrowth CMA<br>number of infections	***0.003***<br>***0.012***<br>***0.008***<br>***0.003***<br>0.057<br>0.063	21.47%	treatment<br>delivery<br>spitting	***0.001***<br>***0.038***<br>0.081	12.08%
**Microbial protein functional classes (KEGG Brite level c)**	allergy father<br>age<br>egg allergy	***0.009***<br>***0.025***<br>***0.017***	12.58%	allergy mother<br>delivery<br>age<br>stool colour	***0.007***<br>***0.012***<br>***0.027***<br>***0.048***	17.22%	treatment<br>SCORAD	***0.003***<br>***0.030***	10.07%
**16s rRNA gene-based taxonomy**	delivery<br>age	***0.003***<br>***0.007***	9.79%	treatment<br>age<br>egg allergy<br>delivery<br>**outgrowth CMA**<br>vomiting<br>stool colour	***0.001***<br>***0.001***<br>***0.005***<br>***0.026***<br>***0.018***<br>***0.021***<br>0.051	32.56%	delivery<br>gaswind<br>treatment<br>stool colour	***0.013***<br>***0.029***<br>***0.039***<br>***0.045***	16.78%
**Protein-based taxonomy**	delivery<br>sibling<br>other allergy	***0.013***<br>***0.011***<br>***0.036***	15.47%	stool colour<br>age	***0.025***<br>***0.039***	9.60%	none	n/a	n/a
**Human proteins**	none	n/a	n/a	allergy mother<br>delivery	***0.016***<br>***0.035***	7.08%	spitting	***0.027***	3.66%

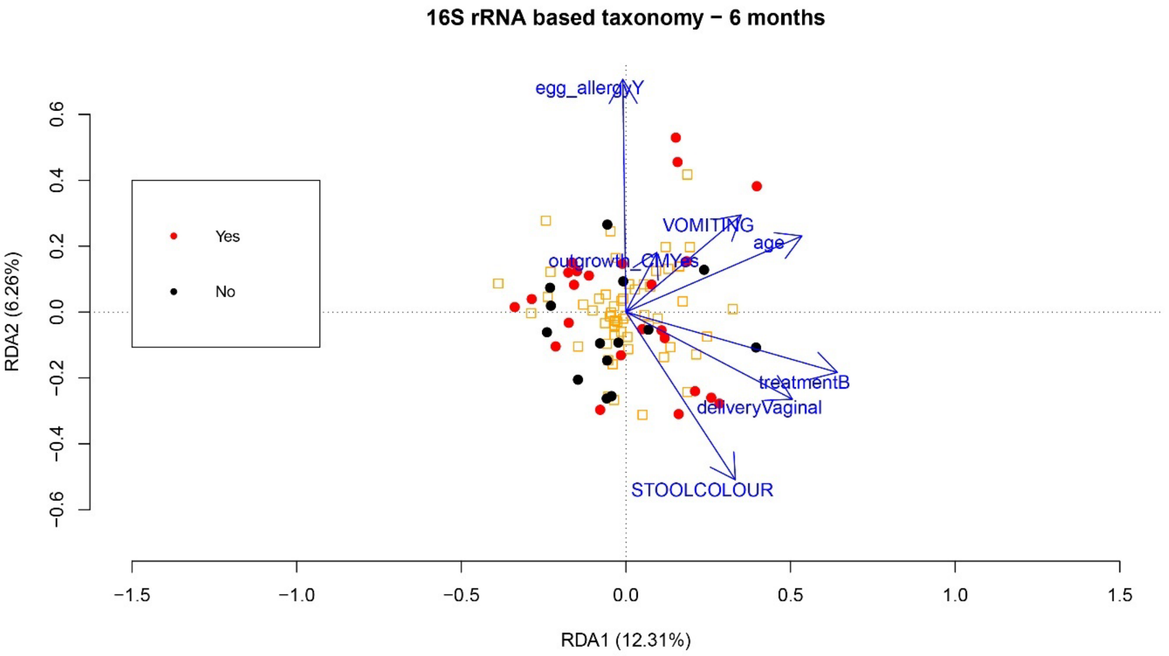

Figure S6. Redundancy analysis (RDA) of the bacterial community in faeces samples for the visit 6 months, coloured by outgrowth of CMA using 16S-rRNA gene based taxonomic profiles at the family level. Arrows indicate features with p-value ≤ 0.1. Red dots: Yes = outgrowth of CMA; black dots: No = no outgrowth of CMA; orange squares: microbial variables (each square represents a family).

Table S30. Results of partial RDA with outgrowth of CMA as explanatory variable, adjusting for other environmental factors. P-value and % variance explained by outgrowth of CMA. Significant results are indicated in bold.

**Type of profile**	**baseline**		**6 months**		**12 months**	
	**p-value**	**%variance explained**	**p-value**	**%variance explained**	**p-value**	**%variance explained**
**Proteome**	0.468	3.54%	0.391	5.24%	0.502	5.04%
**Microbial protein functional classes (KEGG Brite level c)**	0.520	3.29%	0.217	6.15%	0.845	3.32%
**16S rRNA gene-based taxonomy**	0.634	3.05%	**0.044**	**6.37%**	0.087	6.23%
**Protein-based taxonomy**	0.340	3.83%	0.190	6.76%	0.210	6.47%
**Human proteins**	0.842	2.78%	0.505	4.89%	0.479	5.01%

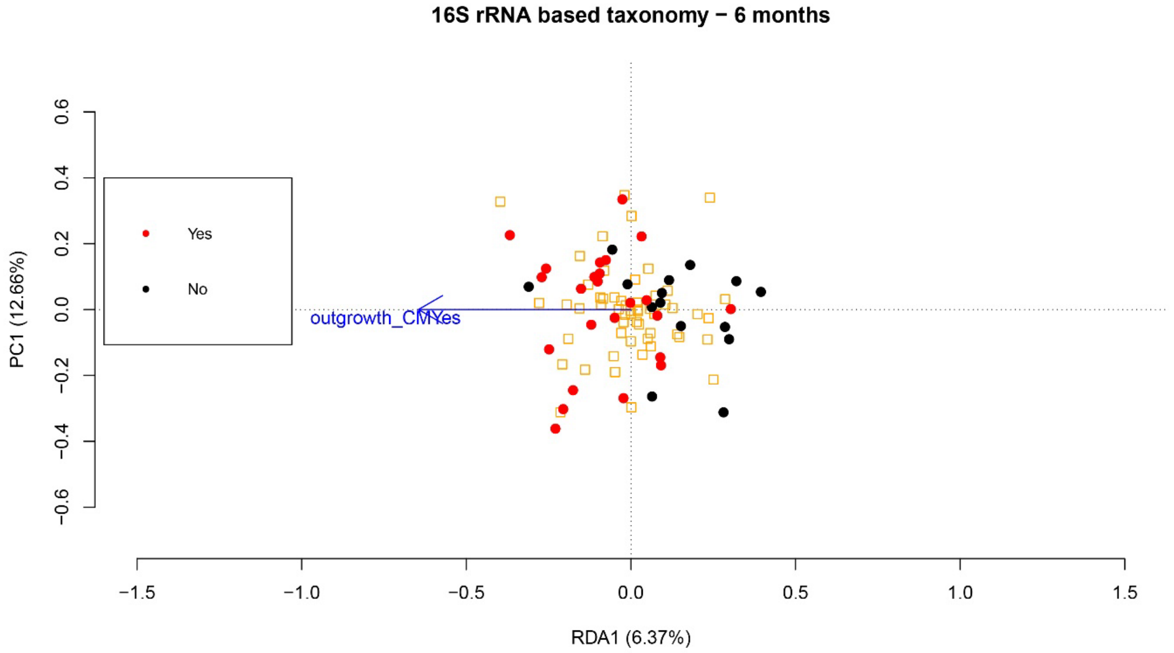

Figure S7. Partial RDA of 16S-rRNA gene based taxonomic profiles at the family level for the visit 6 months, with outgrowth of CMA as explanatory variable and adjusting for other environmental factors. Figure coloured by outgrowth of CMA. Red dots: Yes = outgrowth of CMA; black dots: No = no outgrowth of CMA; orange squares: microbial variables (each square represents a family).

Table S31. Significant (p-value ≤ 0.05, bold italic) and marginally significant (0.05 < p-value ≤ 0.1) results from redundancy analysis (RDA) on microbial proteome profiles and protein-based taxonomic profiles (family level), using human proteins as explanatory variables. adj. p-value: p-value corrected for multiple testing using Benjamini-Hochberg correction.

**Type of profile**	**Baseline**		
	**Variable**	**adj. p-value**	**%variance explained by selected variables**
**Microbial proteome**	Prolactin-inducible protein	***0.001***	11.86%
	Keratin, type II cytoskeletal 8	***0.004***	
	Basic salivary proline-rich protein 2	***0.004***	
	Basic salivary proline-rich protein 3	***0.019***	
**Protein-based taxonomy**	Superoxide dismutase [Cu-Zn]	***0.001***	50.35%
	Immunoglobulin heavy variable 3-15	***0.001***	
	Apolipoprotein D	***0.001***	
	Dihydrolipoyl dehydrogenase, mitochondrial	***0.002***	
	Putative N-acetylated-alpha-linked acidic dipeptidase	***0.006***	
	Fatty acid-binding protein, intestinal	***0.006***	
	Serotransferrin	***0.010***	
	ATP synthase subunit alpha, mitochondrial	***0.005***	
	Pyruvate kinase PKM	0.055	
	Heat shock protein HSP 90-alpha	0.060	
	**6 months**		
	**Variable**	**adj. p-value**	**%variance explained by selected variables**
**Microbial proteome**	POTE ankyrin domain family member E	***0.001***	9.20%
	Elongation factor 1-alpha 1	***0.002***	
	Myeloperoxidase	***0.003***	
**Protein-based taxonomy**	Intelectin-2	***0.001***	44.67%
	Immunoglobulin lambda variable 1-51	***0.001***	
	Myosin-2	***0.003***	
	Meprin A subunit beta	***0.007***	
	Carboxypeptidase B	***0.014***	
	Fatty acid-binding protein, intestinal	***0.013***	
	Alpha-1-antitrypsin	***0.011***	
	Intelectin-1	***0.015***	
	Beta-enolase	***0.035***	
	**12 months**		
	**Variable**	**adj. p-value**	**%variance explained by selected variables**
**Microbial proteome**	Bile salt-activated lipase	***0.001***	15.57%
	Chymotrypsin-C	***0.005***	
	Basic salivary proline-rich protein 3	***0.032***	
	Ferritin heavy chain	***0.026***	
	Transketolase	***0.040***	
**Protein-based taxonomy**	Neutrophil gelatinase-associated lipocalin	***0.001***	88.37%
	Protein S100-A8	***0.001***	
	Alpha-amylase 1	***0.001***	
	Carboxypeptidase A1	***0.001***	
	Putative uncharacterized protein MYH16	***0.001***	
	Transketolase	***0.001***	
	Immunoglobulin heavy constant alpha 2	***0.001***	
	Xaa-Pro aminopeptidase 2	***0.001***	
	Serpin B6	***0.001***	
	Trypsin-1	***0.001***	
	Creatine kinase M-type	***0.001***	
	Ferritin heavy chain	***0.001***	
	Phospholipase A2, membrane associated	***0.001***	
	Tropomyosin alpha-3 chain	***0.001***	
	Alpha-actinin-2	***0.001***	
	Voltage-dependent anion-selective channel protein 1	***0.001***	
	Eukaryotic initiation factor 4A-III	***0.002***	
	Polymeric immunoglobulin receptor	***0.006***	
	Keratin, type II cytoskeletal 8	***0.014***	
	Pancreatic triacylglycerol lipase	***0.007***	
	Immunoglobulin kappa variable 1-6	***0.008***	
	Immunoglobulin kappa constant	***0.014***	
	Ferritin light chain	***0.024***	
	Dihydrolipoyl dehydrogenase, mitochondrial	***0.049***	

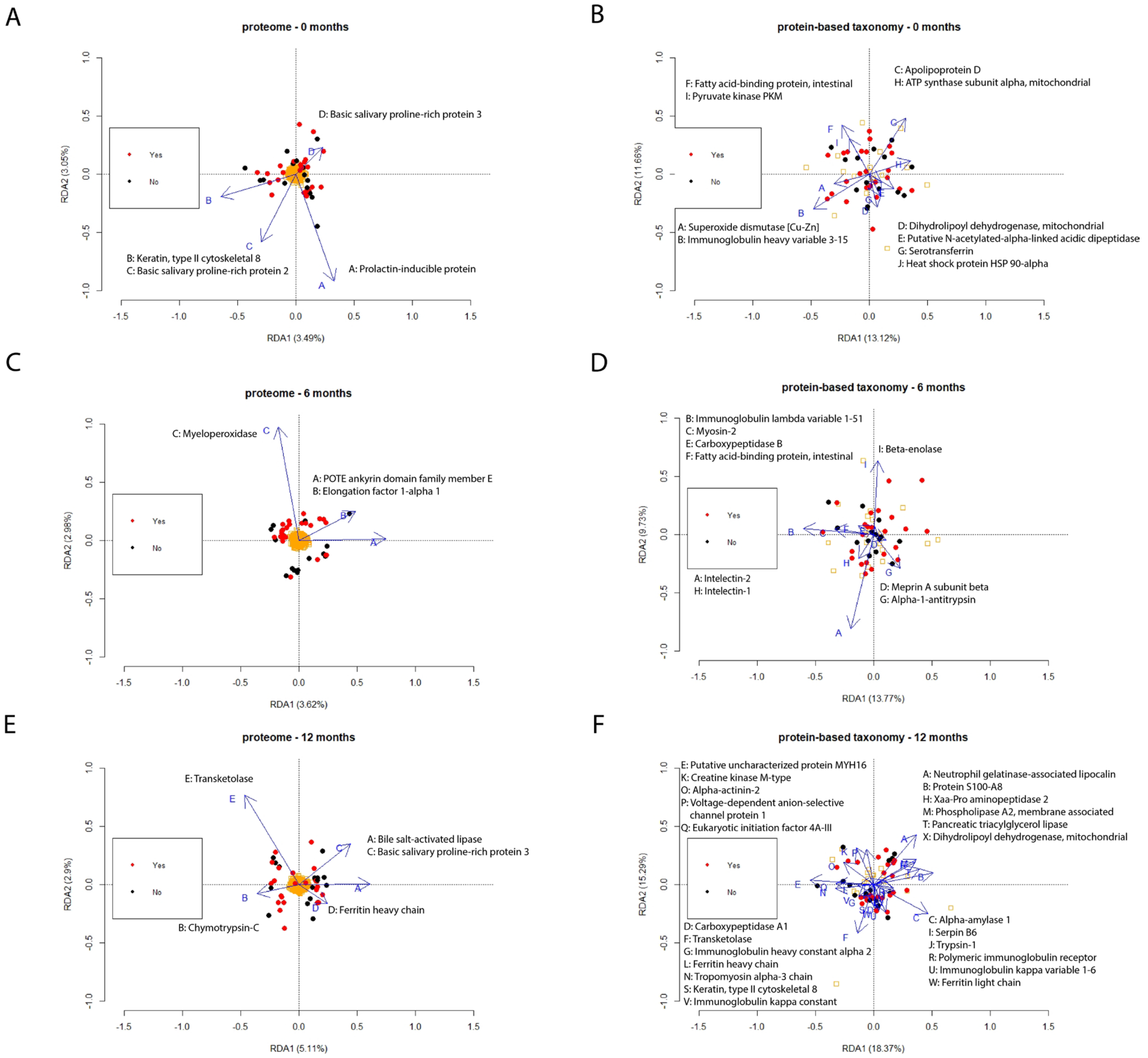

Figure S8. RDA using human proteins as explanatory variables, and coloured by outgrowth of CMA. Red dots: Yes = outgrowth of CMA; black dots: No = no outgrowth of CMA; orange squares: microbial variables (each square represents a microbial protein group (left) or family (right)). (A) RDA of proteome profiles at baseline visit; (B) RDA of protein-based taxonomy profiles (family level) at baseline visit; (C) RDA of proteome profiles at visit 6 months; (D) RDA of protein-based taxonomy profiles (family level) at visit 6 months; (E) RDA of proteome profiles at visit 12 months; (F) RDA of protein-based taxonomy profiles (family level) at visit 12 months.

Table S32. Protein functional classes related to metabolic pathways increased between visits in the group with outgrowth of CMA, but not in the other group as determined by LEfSe analysis. Second column: displays from which families these proteins originate. Third column: displays between which visits the increase was observed. Fourth column: families that show the same behaviour over visits than the protein classes in the first column. Families that increase in the same way as the protein classes (Figure 5) are indicated in bold.

**Protein functional class**	**Families in data set**	**Increase between visits … (Figure 6)**
Selenocompound metabolism	*Bifidobacteriaceae, Lachnospiraceae, Ruminococcaceae*	Baseline and 6 months<br>Baseline and 12 months
Aminoacyl-tRNA biosynthesis	*Bifidobacteriaceae, Lachnospiraceae*	Baseline and 6 months<br>Baseline and 12 months
Amino sugar and nucleotide sugar metabolism	*Bifidobacteriaceae, Lachnospiraceae, Enterococcaceae*	Baseline and 6 months<br>6 months and 12 months
Cysteine and methionine metabolism	*Bacteroidaceae, Bifidobacteriaceae, Ruminococcaceae, Lachnospiraceae, Clostridiaceae*	Baseline and 6 months<br>Baseline and 12 months
Galactose metabolism	*Bifidobacteriaceae, Lachnospiraceae*	Baseline and 6 months
Pentose and glucuronate interconversions	*Bacteroidaceae, Clostridiaceae, Lachnospiraceae, Bifidobacteriaceae*	Baseline and 12 months
Pyruvate metabolism	*Bacteroidaceae, Enterobacteriaceae, Enterococcaceae, Clostridiaceae, Oscillospiraceae, Lachnospiraceae, Prevoltellaceae, Bifidobacteriaceae, Ruminococcaceae, Veillonellaceae,* ***Rikenellaceae***	Baseline and 12 months
Porphyrin metabolism	*Bifidobacteriaceae, Lachnospiraceae*	Baseline and 12 months
Nitrogen metabolism	*Lachnospiraceae, Bacteroidaceae, Clostridiaceae, Ruminococcaceae, Bifidobacteriaceae, Veillonellaceae*	Baseline and 12 months
Fructose and mannose metabolism	*Bacteroidaceae, Streptococcaceae, Bifidobacteriaceae, Lachnospiraceae,* ***Coriobacteriaceae****, Enterococcaceae, Ruminococcaceae, Clostridiaceae*	Baseline and 12 months
Beta-alanine metabolism	*Lachnospiraceae, Veillonellaceae*	Baseline and 12 months
Fatty acid degradation	*Clostridiaceae, Bifidobacteriaceae, Lachnospiraceae, Oscillospiraceae, Veillonellaceae*	Baseline and 12 months
Propanoate metabolism	*Enterobacteriaceae, Clostridiaceae, Lachnospiraceae, Oscillospiraceae, Enterococcaceae, Bacteroidaceae, Bifidobacteriaceae, Veillonellaceae*	Baseline and 12 months
Glycerolipid metabolism	*Lachnospiraceae*	Baseline and 12 months
Streptomycin metabolism	*Bifidobacteriaceae, Lachnospiraceae*	Baseline and 12 months
